# Inositol hexakisphosphate kinases differentially regulate trafficking of vesicular glutamate transporters 1 and 2

**DOI:** 10.3389/fncel.2022.926794

**Published:** 2022-07-22

**Authors:** Haiyan Li, Maia Datunashvili, Reno C. Reyes, Susan M. Voglmaier

**Affiliations:** Department of Psychiatry, School of Medicine, University of California San Francisco, San Francisco, CA, United States

**Keywords:** synaptic vesicle, vesicular glutamate transporter, endocytosis, inositol hexakisphosphate kinase (IP6K), exocytosis

## Abstract

Inositol pyrophosphates have been implicated in cellular signaling and membrane trafficking, including synaptic vesicle (SV) recycling. Inositol hexakisphosphate kinases (IP6Ks) and their product, diphosphoinositol pentakisphosphate (PP-IP_5_ or IP7), directly and indirectly regulate proteins important in vesicle recycling by the activity-dependent bulk endocytosis pathway (ADBE). In the present study, we show that two isoforms, IP6K1 and IP6K3, are expressed in axons. The role of the kinases in SV recycling are investigated using pharmacologic inhibition, shRNA knockdown, and *IP6K1* and *IP6K3* knockout mice. Live-cell imaging experiments use optical reporters of SV recycling based on vesicular glutamate transporter isoforms, VGLUT1- and VGLUT2-pHluorins (pH), which recycle differently. VGLUT1-pH recycles by classical AP-2 dependent endocytosis under moderate stimulation conditions, while VGLUT2-pH recycles using AP-1 and AP-3 adaptor proteins as well. Using a short stimulus to release the readily releasable pool (RRP), we show that IP6K1 KO increases exocytosis of both VGLUT1-and VGLUT2-pH, while IP6K3 KO decreases the amount of both transporters in the RRP. In electrophysiological experiments we measure glutamate signaling with short stimuli and under the intense stimulation conditions that trigger bulk endocytosis. IP6K1 KO increases synaptic facilitation and IP6K3 KO decreases facilitation compared to wild type in CA1 hippocampal Schaffer collateral synapses. After intense stimulation, the rate of endocytosis of VGLUT2-pH, but not VGLUT1-pH, is increased by knockout, knockdown, and pharmacologic inhibition of IP6Ks. Thus IP6Ks differentially affect the endocytosis of two SV protein cargos that use different endocytic pathways. However, while IP6K1 KO and IP6K3 KO exert similar effects on endocytosis after stimulation, the isoforms exert different effects on exocytosis earlier in the stimulus and on the early phase of glutamate release. Taken together, the data indicate a role for IP6Ks both in exocytosis early in the stimulation period and in endocytosis, particularly under conditions that may utilize AP-1/3 adaptors.

## Introduction

Three types of vesicular glutamate transporters (VGLUTs) are responsible for loading synaptic vesicles with glutamate: VGLUTs 1, 2, and 3 (Bellocchio et al., 2000; Takamori et al., 2000; Takamori, 2006). The glutamate transport activities of VGLUT1 and 2 are virtually identical, but these isoforms are differentially expressed and their expression imparts unique properties to synapses (Fremeau et al., 2004b; Weston et al., 2007, 2011). In particular, VGLUT1-expressing synapses exhibit a lower probability of release and synaptic facilitation, while VGLUT2 synapses exhibit a higher probability of release and synaptic depression (Fremeau et al., 2001, 2004a; Voglmaier et al., 2006). These isoform-specific properties are associated with differential interactions of the N- and C-terminal domains of VGLUT1 and VGLUT2 with endocytic proteins such as endophilin (Voglmaier et al., 2006; Weston et al., 2011) and the clathrin adaptor proteins AP-1, AP-2, and AP-3 (Voglmaier et al., 2006; Foss et al., 2013; Santos et al., 2014; Li et al., 2017).

Clathrin adaptor proteins are important in synaptic vesicle (SV) trafficking, acting as coincidence detectors of lipids and protein recognition sequences in membrane proteins (Fei et al., 2008; Mori and Takamori, 2017). At least three SV endocytic pathways employ clathrin adaptors (Heuser and Reese, 1973; Neves and Lagnado, 1999; Richards et al., 2000; Royle and Lagnado, 2010; Santos et al., 2014). At room temperature, AP-2 facilitates vesicle formation from the cell membrane by classical clathrin mediated endocytosis (Takei et al., 1996). AP-1 and AP-3 facilitate vesicle formation from endosomes formed by activity-dependent bulk endocytosis (ADBE) (Faundez et al., 1998; Wu et al., 2007; Cheung and Cousin, 2012). Clathrin independent endocytosis may also use clathrin adaptors (Watanabe et al., 2014; Lopez-Hernandez et al., 2022). Under moderate stimulation conditions, VGLUT1-pHluorin (VGLUT1-pH) recycling depends largely on AP-2, while VGLUT2-pH recycling depends to a larger extent on AP-1 and AP-3 (Foss et al., 2013; Li et al., 2017; Lopez-Hernandez et al., 2022). VGLUT1-pH recycling is faster than VGLUT2-pH, but inhibition of AP-3 accelerates the rate of VGLUT2-pH endocytosis, presumably by redirecting the transporter to the faster AP-2 pathway. However, under prolonged or intense stimulation conditions, VGLUT1 expressing SVs can also recycle by the AP-1/3 pathway (Voglmaier et al., 2006; Clayton et al., 2008; Cheung and Cousin, 2012).

Proper function of AP-3 is important for melanosome trafficking, peptide hormone release, and synaptic transmission (Voglmaier et al., 2006; Newell-Litwa et al., 2007; Silm et al., 2019). Genetic mutation of AP-3 results in aberrant neuronal physiology and behavior in mice, including hyperactivity, seizures, and auditory gating abnormalities (Miller et al., 1999; Noebels and Sidman, 2007). Mice lacking the neuronal AP-3β subunit isoform exhibit decreased asynchronous release and information transfer (Evstratova et al., 2014). While the importance of a functional AP-3 has been demonstrated, the regulation of AP-3 is not fully understood. It is known that inhibiting a casein kinase 1 activity that phosphorylates serine residues in the hinge region of AP-3β hinders SV formation from endosomes (Faundez and Kelly, 2000). The hinge region of the AP-3β subunit can be further modified by pyrophosphorylation of a pre-phosphorylated serine residue by the high-energy inositol phosphate 5-diphosphoinositol pentakisphosphate (PP-IP5 or IP7) (Voglmaier et al., 1996; Saiardi et al., 2004; Azevedo et al., 2009; Werner et al., 2010).

While inositol phosphates (e.g., IP3) and phosphoinositides (e.g., PIP_2_) serve important roles in cell signaling and membrane trafficking, the pleiotropic functions of higher inositol phosphates are just beginning to be elucidated. IP7 is generated by a family of kinases, inositol hexakisphosphate kinase (IP6K) 1, 2, and 3 (Voglmaier et al., 1996; Saiardi et al., 1999, 2001; Schell et al., 1999). IP6K2 is highly expressed in the nucleus (Yong et al., 2015), where it plays a role in apoptosis (Nagata et al., 2005). Several studies implicate IP6K1 in exocytosis and endocytosis of vesicles. IP6K1 modulates endocytic trafficking in yeast, secretion of insulin granules, release of dopamine from PC12 cells, and release of viral particles from mouse embryonic fibroblasts (Luo et al., 2001; Saiardi et al., 2002; Illies et al., 2007; Azevedo et al., 2009). More recently, both IP6 and IP7 have been implicated in the regulation of synaptic signaling and vesicle recycling in hippocampal neurons, perhaps through interaction with synaptotagmin (Yang et al., 2012; Lee et al., 2016; Park et al., 2020). Indeed, IP6K1 may play a fundamental role in behavior (Chakraborty et al., 2014; Kim et al., 2020).

Inositol polyphosphate balance is dynamic and sensitive to cellular levels of ATP and ADP (Glennon and Shears, 1993; Menniti et al., 1993; Voglmaier et al., 1996; Wilson et al., 2013). SV recycling and the maintenance of SV pools are major consumers of cellular energy (Rangaraju et al., 2014; Pulido and Ryan, 2021). IP7 directly and indirectly regulates glycogen synthase kinase 3 (GSK-3), a key regulator of metabolic and SV bulk endocytosis pathways (Barker et al., 2009; Chakraborty et al., 2010, 2014; Clayton et al., 2010). IP6K1 binds GSK-3α and β, stimulating their catalytic activities, and affecting behavior (Chakraborty et al., 2014). IP6K1 also indirectly activates GSK-3 by inhibiting Akt affecting glucose homeostasis and insulin sensitivity (Chakraborty et al., 2010). The known effects of IP6K and IP7 on GSK-3 and AP-3 directed our investigations to test conditions under which SV recycling is known to be affected by GSK-3 or AP-3. Because VGLUT2-pH recycling is sensitive to inhibition of AP-3, we sought to determine whether VGLUT2 recycling can be regulated by IP6Ks as well. We also test how IP6Ks affect SV recycling and glutamate release under the intense stimulation conditions that induce recycling by the AP-1/3 and GSK-3-dependent bulk endocytosis pathway in VGLUT1 synapses (Clayton et al., 2008; Cheung and Cousin, 2012). We investigate these effects using pharmacologic inhibition, shRNA knockdown, and well-characterized IP6K1 and IP6K3 knockout mice (Bhandari et al., 2008; Prasad et al., 2011; Chakraborty et al., 2014; Fu et al., 2015; Chanduri et al., 2016; Moritoh et al., 2016; Rojas et al., 2019).

## Materials and methods

### Reagents

*N*^2^-(*m*-trifluoromethylbenzyl) *N*^6^-(*p*-nitrobenzyl)purine (TNP), and bafilomycin A1 were acquired from Calbiochem (MilliporeSigma). CNQX (6-cyano-7 nitroquinoxaline-2,3-dione) and CPP (3-(2-carboxypiperazin-4-yl) propyl-1-phosphonic acid) were obtained from Tocris Bioscience. Antibodies against IP6K1, IP6K3, Neurofilament-heavy (NF-H; RMO-24) and MAP2 were purchased from Genetex, Assay Biotech, Novus Biologicals and Sigma-Aldrich, respectively. Secondary antibodies conjugated to Cy2, Cy3, or Cy5 were from Jackson ImmunoResearch. All other chemicals are from Sigma-Aldrich or Fisher Scientific. Cell culture reagents are from Life Technologies unless otherwise noted.

### Molecular biology

Mouse and human IP6K1 (kind gift of A. Chakraborty, St. Louis University) were subcloned into a pCAGGS vector by standard techniques, and the sequence confirmed.

### RNA interference knockdown

Lentiviral constructs expressing shRNA to rat IP6K1 (IP6K1 shRNA, 5′-TCAACCTGGTAGCCTACCCTT-3′) and IP6K3 (5′-CCATCTCGGCCTGGTTGCCAA-3′, sequences provided by A. Chakraboty, St. Louis U) (Rao et al., 2014b; Fu et al., 2015) were made in pFHUBW vector (gift of Roger Nicoll, UCSF), a variant of pFHUGW containing the monomeric blue fluorescent protein mTagBFP in place of GFP (Lois et al., 2002). The resulting pFHUBW vector was cotransfected along with two packaging plasmids (pVSV-G and psPAX2) into HEK293T cells using FuGENE HD (Promega), as previously described (Foss et al., 2013). HEK293T cells were grown in UltraCULTURE serum-free media (Lonza) and supplemented with 1 mM sodium pyruvate, 0.075% bicarbonate and 2 mM GlutaMax. At approximately 16 h after transfection, 10 μM sodium butyrate was added to the culture media, and at 40 h after transfection, the culture media was collected, and viral particles concentrated by centrifugation through a 20% sucrose/PBS gradient at 80,000 × *g* for 2 h at 4°C. Viral particles were resuspended in neuronal culture media supplemented with 4 μg/ml Polybrene (hexadimethrine bromide) (Zhang et al., 2010).

### Immunofluorescence

For immunostaining, cells were fixed in 4% paraformaldehyde for 5 min, permeabilized and blocked in phosphate buffer saline (PBS) containing 0.02% saponin, 1% fish gelatin, and 5% bovine serum albumin (BSA) and then incubated with rabbit anti-IP6K1, mouse anti-MAP2 and chicken anti-NFH, or rabbit anti-IP6K3, mouse anti-MAP2 and chicken anti-NFH. After brief washes of the primary antibodies above, the cells were incubated with secondary antibodies conjugated to Cy2, Cy3, or Cy5. Cells were imaged using confocal laser microscopy (Leica TCS SP5). To confirm specific knockdown of IP6Ks, IP6K expression was measured by immunofluorescence in HEK293 cells and neurons (Li et al., 2005). Rat IP6K1 or 3 was inserted into the pCMV-myc vector (Clontech) and transfected using Lipofectamine 2000 (Thermo Fisher) in HEK293 cells grown on glass coverslips. Cells were co-transfected with either control FHUBW vector, or FHUBW containing the shRNA sequence against rat IP6K1 and IP6K3. After 3 days, cells were processed for immunocytochemistry and probed for IP6K expression using rabbit anti-IP6K antibodies and appropriate secondary antibodies in cells expressing BFP. To confirm specific knockdown of endogenous IP6K in neurons, rat hippocampal neurons grown on glass coverslips were infected with virus containing FHUGW alone or FHUGW containing shRNA against IP6Ks at DIV7, then fixed on DIV14, and stained with IP6K antibodies and appropriate secondary antibodies. The immunofluorescence intensity of antibody staining was measured by epifluorescence microscopy in manually selected ROIs centered over boutons expressing BFP (Foss et al., 2013).

### Primary neuronal culture and transfection

All work with animals was conducted under the supervision and guidance of the Institutional Animal Care and Use Committee of the University of California, San Francisco. Timed-pregnant Sprague-Dawley rats were purchased from Charles River Laboratories. IP6K KO mice were bred as heterozygotes and postnatal day 0 to 1 (P0–P1) pups genotyped the day of culture (Bhandari et al., 2008). For rat neuronal cultures, hippocampi from embryonic day E19–20 rat pups of either sex were dissected and dissociated as previously described (Li et al., 2011). Neurons were transfected using the Basic Neuron SCN Nucleofector kit as per manufacturer’s directions (Lonza). Neurons transfected *via* nucleofection express similar and moderate levels of protein (Li et al., 2005). Cells were maintained in Neurobasal media supplemented with 1% heat inactivated fetal bovine serum (FBS), 2% B-27, 2 mM GlutaMax, 15 mM NaCl, and 10 μg/ml Primocin antibiotic (Lonza), and 5-fluoro-2′-deoxyuridine (10 μM final concentration) was added at 3–5 days *in vitro* (DIV) as a mitotic inhibitor to control glial growth. For RNAi knockdown experiments, cells were infected at DIV7 and imaged at DIV14–16. For mouse neuronal cultures, hippocampi from P0-P1 pups of either sex were dissected and dissociated after confirmation of genotype by PCR from tail DNA. Mouse tissues were digested using 200U papain (Worthington) in HBSS buffer containing 0.2 mg/ml L-cysteine (Fluka), 500 μM EDTA, 1 mM CaCl_2_, 3 mM NaOH, and digestion was terminated with 2.5 mg/ml soybean trypsin inhibitor (Worthington), and 2.5 mg/ml bovine serum albumin (BSA) in minimum essential media (MEM, Thermo Fisher) growth solution. MEM growth solution consists of MEM with 5% FBS, 2% B-27, 1% Glutamax, 0.1% MITO+ Serum Extender (BD Biosciences), and 21 mM glucose (Sigma-Aldrich). Mouse neurons were also transfected using the Basic Neuron SCN Nucleofector kit, maintained in Neurobasal media as described above, and 5-fluoro-2′-deoxyuridine (10 μM final concentration) was added at DIV5-7.

### Live cell imaging

Rat and mouse neurons were imaged at DIV14–19. Live cell imaging was performed as described previously (Voglmaier et al., 2006). Coverslips with transfected hippocampal neurons were mounted in a rapid switching, laminar-flow perfusion and stimulation chamber (Warner Instruments, Holliston, MA, United States) on an inverted epifluorescence microscope (Nikon, Melville, NY) and imaged at room temperature using a 63X oil objective (NA = 1.4). Cells were imaged in modified Tyrode’s solution pH 7.4 (in mM: 119 NaCl, 10 HEPES, 30 glucose, 2.5 KCl, 2 CaCl_2_, MgCl_2_), and containing 10 μM of the glutamate receptor inhibitors CNQX and CPP. Electrical stimulation to elicit action potentials was applied using an A310 Accupulser (WPI) at 10–80 Hz with 1 ms bipolar current pulses through platinum-iridium electrodes, to yield fields of 5–10 V/cm across the chamber. Cells were illuminated using a Xenon lamp (Sutter Instruments) with a 470/40-nm excitation and a 525/50-nm emission filter for GFP (Chroma). Images were acquired on a QuantEM CCD camera (Photometrics) exposing each fluorophore for 100–300 ms for images collected every 1 or 3 s. Metamorph software was used to control data collection and to perform offline analysis (Molecular Devices). The total pool size was determined using Tyrode’s solution with 50 mM NH_4_Cl (NaCl reduced to 50 mM). To measure exocytosis alone, cultures were incubated in modified Tyrode’s solution containing 0.5–1 μM bafilomycin A1 for 30 s before imaging and during the imaging period. For TNP and BfA experiments, cultures were pretreated in complete Neurobasal media containing 10 μM TNP for 2 h and/or 10 μg/ml BfA for 30 min at 37°C before imaging in modified Tyrode’s solution containing the same concentration of TNP and/or BfA.

FM dye experiments were performed essentially as described (Cheung et al., 2010). Cultured hippocampal neurons were incubated in modified Tyrode’s solution containing 10 μM FM1–43 and stimulated at 80 Hz for 10 s to load the dye. Cells were washed for 2 min at 6 ml/min in modified Tyrode’s solution without FM1–43, then FM dye was unloaded by stimulation at 30 Hz for 2 s to release the readily releasable pool. The recycling pool was released with three subsequent trains of 40 Hz 10 s stimulation. Fluorescent time courses from 60 to 153 boutons from 5 to 7 coverslips from 2 independent cultures were normalized to initial fluorescence and averaged.

### Imaging data analysis

As described previously (Voglmaier et al., 2006; Li et al., 2011), Metamorph software was used to quantify the average fluorescence of ROIs at synaptic sites at manually selected 4 × 4 pixel boxes placed over the center of boutons. The average fluorescence of three 4 × 4 pixel ROIs without cellular elements was subtracted as background. Baseline values from the first 5 frames (before stimulation) were averaged as initial fluorescence F_0_, and the dynamics of fluorescence intensity expressed as fractional change (ΔF) over initial fluorescence. For normalized measurements, the average pHluorin fluorescence over individual boutons was normalized to either the peak fluorescence in each trace, or the total fluorescence as visualized by application of modified Tyrode’s solution containing 50 mM NH_4_Cl to alkalinize all synaptic compartments. Fluorescence measurements from at least 30 boutons per coverslip were averaged and the means from 5 to 12 coverslips from at least two independent cultures were averaged, with the number of coverslips treated as independent units of observation (n) (Onoa et al., 2010). Data presented as means ± SEM. Significance of differences between groups was assessed by two-tailed, unpaired *t*-test and one-way ANOVA where appropriate, with significance at *p* < 0.05 (GraphPad Prism).

To measure the rate of exocytosis and to determine the total amount of transporter that underwent exocytosis, cells were imaged in modified Tyrode’s medium containing bafilomycin A1. The fraction of transporter that undergoes exocytosis (recycling pool, RP) was measured as the fraction of the total pool that undergoes exocytosis in response to 10 Hz 90 s stimulation (Foss et al., 2013; Santos et al., 2013). To determine the decline from peak during stimulation [Δ(ΔF/F_0_)], the fluorescence recorded at the last time point of stimulation (60 s) was subtracted from peak fluorescence, and the difference expressed as a percentage of peak fluorescence. For measurements of endocytosis after stimulation, the time course of fluorescence decay at each bouton after the initial 3 s was fit with a single exponential (GraphPad Prism) (Balaji et al., 2008).

### Slice preparation

For electrophysiological *in vitro* whole cell patch clamp experiments, 4–6 week old IP6K1 and IP6K3 KO mice of either sex and wild type (WT) littermates were decapitated, brains removed and immersed in ice-cold sucrose saline containing (in mM): sucrose, 234; NaHCO_3_, 25; KCl, 2.5; NaH_2_PO_4_, 1.25; MgSO_4_, 10; CaCl_2_, 0.5; dextrose, 10; pH was adjusted to 7.4 by carbogen (95% O_2_, 5% CO_2_ gas mixture). The osmolarity was 300–310 mOsm. Coronal hippocampal slices of 400 μm thickness were prepared on a vibratome (Leica, VT1200S, Germany) to study the Schaffer collateral-CA1 pathway (Papaleonidopoulos et al., 2017; Xiong et al., 2017). Thalamostriatal slices were prepared based on a modification of a previously described procedure (Smeal et al., 2007). In brief, the brain was glued on a triangular agar block with the olfactory bulb facing down and 30° angles cut. Slices of 370–400 μm thickness were prepared on the vibratome. Slices were transferred to a recovery chamber in a solution (in mM): NaCl, 125; KCl, 2.5; NaH_2_PO_4_, 1.25; NaHCO_3_, 25; MgSO_4_, 2; CaCl_2_, 2; dextrose, 10; pH was equilibrated with carbogen at 7.4 and incubated approximately 20–30 min at 34°C, then at room temperature until recordings started.

### *In vitro* patch clamp electrophysiology

After 40–60 min of recovery, individual slices were placed into a recording chamber and perfused continuously with oxygenated ACSF containing (in mM): NaCl, 125; KCl, 2.5; NaH_2_PO_4_, 1.25; NaHCO_3_, 25; MgSO_4_, 2; CaCl_2_, 2; dextrose, 25. All recordings were done at 32–34°C. Slices were visualized under an upright microscope (Olympus, BX51WI). For cell identification, a 40× water-immersion objective (Olympus) and IR-2000 (DAGE-MTI) camera was used. Whole-cell patch clamp recordings were made from the soma of CA1 pyramidal neurons or striatal medium spiny neurons (MSNs) using an Axopatch 200B amplifier (Molecular Devices, United States). Cell capacitance and access resistance were monitored throughout the experiments. Signals were acquired with a DigiData-1440A digitizer controlled by pCLAMP-11 software (Molecular Devices, United States). Signals were sampled at 10 kHz and low-pass filtered at 2 kHz. Borosilicate glass pipettes (3.5–4.2 MΩ) were pulled with a horizontal puller (P-1000, Sutter Instruments).

Spontaneous (sEPSCs) and miniature (mEPSCs) excitatory currents were recorded from the striatum. EPSCs were isolated from the GABA-ergic transmission by applying 50 μM GABA_*A*_ blocker picrotoxin in the ACSF. To capture mEPSCs, voltage gated Na^+^ channels were blocked with 1 μM TTX. These recordings were done from the slices where both cortical and thalamic connections were intact. Patch pipettes were filled with K-gluconate intracellular solution (in mM): K-gluconate, 88; K_3_-citrate, 20; NaCl, 10; HEPES, 10; MgCl_2_, 1; CaCl_2_, 0.5; BAPTA, 3; Mg-ATP, 3; Na_2_GTP, 0.5. pH to 7.25 was maintained with KOH. Osmolality was set to 300–305 mOsm/kg. EPSCs were measured from a holding membrane potential of −70 mV. The whole cell voltage-clamp protocol contained 30 s steps, repeated 10 times. Recordings lasted 5 min.

For paired-pulse and train stimulation experiments, EPSCs were evoked (eEPSCs) using a bipolar stimulation electrode (from FHC or WPI). In hippocampus, EPSCs were evoked by stimulation of Schaffer collaterals and evoked currents were recorded from CA1 pyramidal neurons. For thalamostriatal slices, to avoid the excitation of both cortical and thalamic fibers, the cortex was removed. To evoke thalamostriatal currents, a stimulation electrode was placed in the thalamus, close to the medial border of the thalamic reticular nucleus (Smeal et al., 2007). EPSCs were evoked with the intensities just sufficient to measure reliable currents. The amplitude of thalamostriatal currents range between 50 and 100 pA. Slices were continuously bathed in ACSF in the presence of 50 μM picrotoxin. To block postsynaptic K^+^ channels, a cesium-based intracellular solution was used for these experiments (in mM): CsMeSO3, 135; CsCl, 6; HEPES, 10; phosphocreatine, 10; EGTA, 0.6; Mg-ATP, 3; Na-GTP, 0.5; QX314-Br, 2. pH was set to 7.35 by CsOH and osmolarity to 305–310 mOsm/kg.

Two short pulses were delivered to conduct paired-pulse recordings. Different interstimulus intervals (ISI) were compared: 25, 50, 100, and 1000 ms. For each ISI 10 individual responses were measured and synapses were allowed to recover 60 between stimulations. Paired-pulse ratios (PPR) were calculated by dividing the second response by the first response (A2/A1). For input-output curves, 30, 50, and 70 pA current intensities were examined and eight trials at the same stimulus intensity were averaged. To examine the frequency-dependent properties of hippocampal and thalamostriatal synapses, trains of ten pulses were delivered at varying frequencies 10, 20, and 40 Hz. All recordings were repeated up to 5 times. Again, the test stimuli were given every 60 s. Average EPSC values from different recordings were normalized to the first EPSC peak amplitude. To estimate RRP size and release probability (P_*r*_), 100 pulses at 40 Hz were applied to Schaffer collateral synapses, allowed to recover for 7 min, and rechallenged with 100 pulses at 40 Hz and current amplitudes measured. Currents were normalized to the first EPSC amplitude. To measure the recovery of glutamate release after high frequency prolonged stimulation in VGLUT1 and VGLUT2 synapses, baseline currents were recorded at 1 Hz (5 s) and then 20 Hz 300 (15 s) pulses were delivered at Schaffer collaterals and thalamostriatal synapses. After synaptic depression, currents recovered at 1 Hz for 150 s. Data analyses were done in Clampfit software (Molecular Devices, United States). Data presented as means ± SEM. Significance of differences between groups was assessed by two-tailed, unpaired *t*-test at *p* < 0.05 and for more than two variables, by ANOVA with Tukey’s post-test where appropriate (GraphPad Prism). Numbers given in text (n) refer to numbers of neurons recorded from at least 3 different animals for each condition.

## Results

### Endocytosis of VGLUT2-pH is accelerated by pharmacological inhibition of inositol hexakisphosphate kinases

To determine the effect of IP6Ks on SV recycling by AP-3, we use an optical reporter of VGLUT2, which relies to a large extent on AP-3 for endocytosis (Li et al., 2017). The closely related isoform VGLUT1, on the other hand, relies predominantly on AP-2 for its recycling, so we use a VGLUT1 optical reporter for this recycling pathway (Voglmaier et al., 2006; Foss et al., 2013; Lopez-Hernandez et al., 2022). To monitor exocytosis and endocytosis of vesicular glutamate transporters in individual synaptic boutons, we transfected cultured hippocampal neurons with fusions of the transporters with pHluorin, a green fluorescent protein shifted in its pH sensitivity (Voglmaier et al., 2006; Li et al., 2017). The fluorescence of the pHluorin fusions is quenched at the low pH (5.5) of SVs. Exocytosis induced by action potential (AP) stimulation exposes pHluorin to the higher pH (7.4) of the external solution, increasing fluorescence. Re-acidification of the vesicle after endocytosis again quenches the fluorescence, thus decreases in fluorescence measures endocytosis. We have previously shown that activity-dependent unloading of the styryl dye FM4–64 from SVs is not affected by expression of VGLUT1-pH or VGLUT2-pH, indicating that the tagged transporters do not perturb general features of the vesicle cycle (Voglmaier et al., 2006; Li et al., 2017).

To induce recycling of SVs by AP-1 and AP-3, we used a high intensity stimulation protocol, inducing action potentials with electrical stimulation at 40 Hz for 60 s (Clayton et al., 2008; Cheung and Cousin, 2012). We previously found that the rate of endocytosis of VGLUT2-pH after 40 Hz 60 s stimulation is significantly slower than VGLUT1-pH (Li et al., 2017). Inhibition of AP-1 and AP-3 function by brefeldin A (BfA) mitigates the differences between VGLUT1-pH and 2-pH endocytic rates. Further, shRNA knockdown of AP-3 speeds endocytosis of VGLUT2-pH, presumably by blocking a slower AP-3 recycling pathway, redirecting VGLUT2-pH to a faster AP-2 pathway (Voglmaier et al., 2006).

**FIGURE 1:**
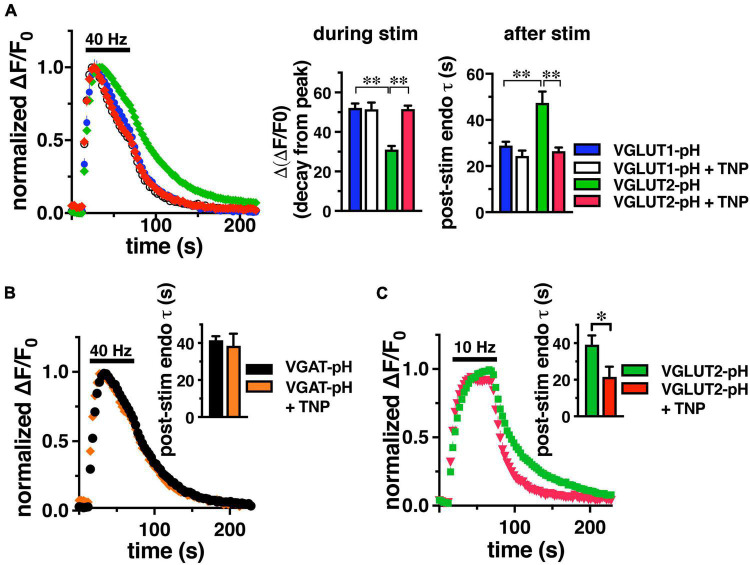
IP6K inhibition accelerates the rate of VGLUT2-pH endocytosis. **(A)** The time course of fluorescence changes (ΔF/F_0_) at synaptic boutons of cultured rat hippocampal neurons expressing VGLUT1-pH or VGLUT2-pH stimulated at 40 Hz for 60 s, normalized to peak fluorescence, exhibit increases and decrease in fluorescence intensity consistent with exocytosis followed by endocytosis. Treatment with 10 μM TNP for 2 h increases the rate of endocytosis of VGLUT2-pH (red) compared to vehicle (green). VGLUT1-pH recycling (blue) is not affected by TNP (white). *Middle panel:* the extent of fluorescence decay from peak fluorescence [Δ(Δ*F*/F_0_)] during stimulation is smaller for VGLUT2 (green) than VGLUT1 (blue) (^**^p < 0.01). Treatment with TNP has no effect on the Δ(Δ*F*/F_0_) of VGLUT1 (white), but significantly increases Δ(Δ*F*/F_0_) for VGLUT2 (red, ^**^p < 0.01). *Right panel:* the rate of post-stimulus endocytosis of VGLUT2-pH in vehicle-treated neurons (green) is significantly slower than VGLUT1-pH. TNP treatment speeds VGLUT2-pH endocytosis to a rate comparable to VGLUT1-pH (^**^p < 0.01, *t*-test). **(B)** TNP treatment has no effect on endocytosis of the AP-2 cargo VGAT-pH. **(C)** TNP accelerates VGLUT2-pH endocytosis after a more moderate 10 Hz 60 s stimulus (*p < 0.05, *t*-test). Data are means ± SEM of ΔF/F_0_ normalized to total fluorescence, *n* = 5–18 coverslips (cs) from at least two independent cultures with at least 35 synapses analyzed per cs.

Since IP6K pyrophosphorylates AP-3, we tested whether inhibition of IP6K affects VGLUT2-pH recycling. Cultured rat hippocampal neurons were transfected with either VGLUT1-pH or VGLUT2-pH to compare the kinetics of recycling of these reporters, as described previously (Foss et al., 2013). We incubated neurons with a selective inhibitor of IP6Ks, N(2)-(m-(trifluoro-methyl)-benzyl) N(6)-(p-nitrobenzyl)purine (TNP) or vehicle control (DMSO). Incubation in 10 μM TNP for 2 h has been shown to decrease IP7 levels by 90% in cell culture (Padmanabhan et al., 2009). Consistent with previous findings, VGLUT1-pH undergoes endocytosis significantly faster than VGLUT2-pH stimulated at 40 Hz for 60 s (Figure 1). During stimulation, the decline in fluorescence from peak levels [Δ(Δ*F*/F_0_)] reflects net endocytosis, and is smaller for VGLUT2-pH (green, 31.07 ± 1.81%) than VGLUT1-pH (blue, 52.12 ± 2.24%, ^**^p < 0.01, ANOVA) (Figure 1, middle panel). The rate of endocytosis after the stimulus is also slower for VGLUT2-pH (green, τ_*decay*_ = 47.38 ± 4.88 s) than VGLUT1-pH (blue, τ = 28.97 ± 1.66 s, ^**^p < 0.01, ANOVA) (Figure 1, right panel).

Interestingly, treatment with TNP accelerates VGLUT2-pH recycling after intense stimulation, while the kinetics of VGLUT1-pH are unaffected, similar to inhibition of AP-3 (Li et al., 2017; Figure 1A). During stimulation, the decline of fluorescence from the peak of VGLUT2-pH in neurons treated with TNP (red, 51.60 ± 1.76%) is accelerated to a rate similar to VGLUT1-pH (+ TNP, white, 51.45 ± 3.43%), and significantly different from VGLUT2-pH control (green, 31.07 ± 1.81%, ^**^p < 0.01, ANOVA) (Figure 1, middle panel). Similarly, the post-stimulus rate of endocytosis of VGLUT2-pH is significantly faster after treatment with TNP (red, τ = 26.49 ± 1.54 s), comparable to VGLUT1-pH in the absence or presence of TNP (VGLUT1-pH + TNP, white, τ = 24.37 ± 2.30 s, ^**^p < 0.01, ANOVA) (Figure 1A, right panel).

An optical reporter of the vesicular GABA transporter VGAT-pH recycles predominantly by AP-2 (Santos et al., 2013, Lopez-Hernandez et al., 2022). Similar to VGLUT1, the rate of VGAT-pH is not affected by TNP (orange, τ = 38.53 ± 6.56 s), compared to control (black, τ = 41.52 ± 2.19 s) Data are means ± SEM from 44 to 110 boutons from 4 to 5 coverslips (Figure 1B). VGLUT2-pH also recycles by an AP-1/3 dependent pathway upon more moderate stimulation (Li et al., 2017), so we tested the effect of TNP on VGLUT2-pH recycling after 10 Hz 60 s stimulation. Under these conditions in mouse hippocampal neurons, TNP treatment increases the rate of VGLUT2-pH endocytosis (red, τ = 21.56 ± 5.61 s), compared to control (green, τ = 39.13 ± 5.05 s, *p < 0.05) (Figure 1C). Thus, pharmacological inhibition of IP6Ks speeds endocytosis of SV cargo that uses AP-3 (VGLUT2), but does not affect AP-2 cargo (VGLUT1, VGAT).

### Subcellular localization of inositol hexakisphosphate kinase subtypes in dissociated hippocampal neurons

**FIGURE 2:**
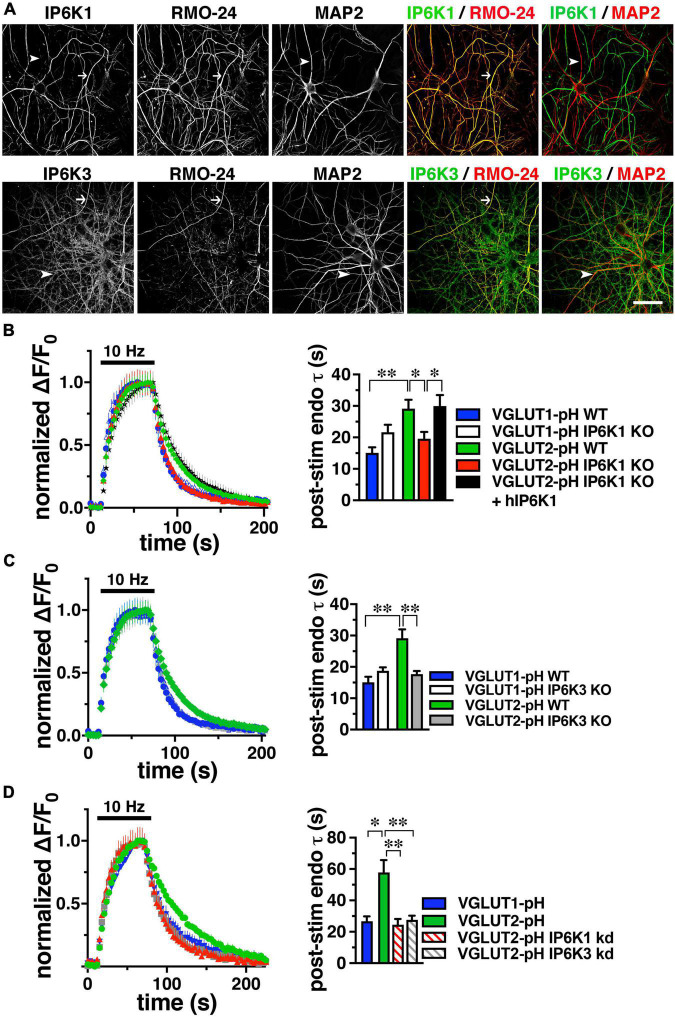
IP6K1 or IP6K3 deletion accelerates VGLUT2-pH endocytosis. **(A)** Co-localization of endogenous IP6K1 and IP6K3 (green) in DIV14 mouse hippocampal neurons with the axonal neurofilament marker RMO-24, and the dendritic marker MAP2 (red). Arrows indicate IP6K1 and 3 colocalization with RMO-24 (yellow in merge panels). Arrowheads indicate colocalization of IP6K3, but not IP6K1, with MAP2. **(B)** Time course and quantification (τ) of normalized fluorescence changes (ΔF/F_0_) of VGLUT-pHs in response to 10Hz 60 s stimulation in WT and IP6K1 KO neurons. **(C)** Fluorescence changes of VGLUT-pHs in WT and IP6K3 KO neurons. **(D)** Fluorescence changes in neurons transfected with VGLUT2-pH and infected with lentivirus containing empty vector or shRNA to IP6K1 or IP6K3. Data are means ± SEM from at least 35 synapses from 5 to 8 cs from at least three independent cultures, *p < 0.05, ^**^p < 0.01, ANOVA.

Because neurons may express all three IP6K isoforms (Chakraborty et al., 2014; Fu et al., 2015), to determine which isoforms of IP6K underlie the effect of TNP on VGLUT2-pH endocytosis, we first determined which IP6K isoforms are expressed in axons. Although IP6K1 has been shown to affect SV recycling (Lee et al., 2016; Park et al., 2020) and interact with GRAB (guanine exchange factor for Rab3a), a protein that may interact with SVs (Luo et al., 2001), the subcellular location of IP6Ks in neurons has not been extensively examined. To verify reactivity of several commercial antibodies against rat IP6K1, 2, and 3, we generated constructs for *myc*-tagged rat IP6Ks by PCR, employing primers designed for known cDNA (IP6K1 and 2) and genomic (IP6K3) sequences (NCBI) and expressed them in HEK293 cells by lipofection. Fixed cells exhibited colocalization of mouse anti-*myc* (Covance) with rabbit anti-human IP6K1 (Genetex) (Ghoshal et al., 2016), goat anti-human IP6K2 (Santa Cruz) (Sahu et al., 2020), or rabbit anti-human IP6K3 (Assay Biotech) antibodies (Supplementary Figure 1). To determine the endogenous localization of the three IP6K subtypes within neurons, we counterstained with established axonal (acetylated tubulin, RMO-24, BD) and dendritic (MAP2, Sigma) markers in fixed rat primary hippocampal neurons (Figure 2A). Punctate staining for IP6K1 is found in neuronal cell bodies, nuclei and processes; staining for IP6K3 is predominantly in processes. Consistent with its role in cell death, IP6K2 is generally confined to the nucleus and cell body (Rao et al., 2014a). IP6K1 colocalizes more extensively with RMO-24 in axons than MAP2 in dendrites, while IP6K3 colocalizes with both markers extensively. Indeed, in cerebellar neurons IP6K3 has been shown to colocalize with dendritic markers and play a role in the arborization of dendrites (Fu et al., 2015). Thus, IP6K1 and 3 both localize presynaptically to axons where they could mediate modulation of VGLUT recycling.

### VGLUT2-pH endocytosis is accelerated by deletion of IP6K1 or IP6K3

To determine the effect of genetic deletion of IP6Ks, primary hippocampal cultures from IP6K1 and 3 knockout (KO) mice (kind gift of Solomon Snyder, Johns Hopkins) (Bhandari et al., 2008; Fu et al., 2015) were transfected with VGLUT1- or 2-pH, stimulated at 10 Hz for 60 s, and the time course of fluorescence responses compared, as described above. Neuronal cultures from IP6K1 KO mice have been shown to contain approximately 50% of control levels of IP7 (Chakraborty et al., 2014). The post-stimulus fluorescence decay measuring VGLUT1-pH endocytosis in IP6K1 KO neurons (white, τ = 21.63 ± 2.43 s) is not significantly different from that in wild type (WT) littermate controls (blue, τ = 15.07 ± 1.81 s) (Figure 2B). However, in the absence of IP6K1, VGLUT2-pH undergoes endocytosis at a faster rate (red, τ = 19.56 ± 2.16 s) compared to VGLUT2-pH endocytosis in WT cultures (green, τ = 29.17 ± 2.79 s, *p < 0.05, ANOVA). Rescue by transfection of a human IP6K1 construct into IP6K1 KO cultures slows the rate of VGLUT2-pH endocytosis to the rate of VGLUT2-pH in WT (black, τ = 29.91 ± 3.52 s, *p < 0.05, ANOVA) (Figure 2B, right panel). Similarly, VGLUT1-pH endocytosis in IP6K3 KO neurons (white, τ = 18.75 ± 1.11 s) is not significantly different from that in WT (blue) (Figure 2C). In the absence of IP6K3, however, VGLUT2-pH undergoes endocytosis at a faster rate (gray, τ = 17.70 ± 0.98 s) compared to VGLUT2-pH endocytosis in WT cultures (^**^p < 0.01, ANOVA). Thus, knockout of either IP6K1 or IP6K3 speeds VGLUT2-pH endocytosis.

### VGLUT2-pH endocytosis is accelerated by knockdown of IP6K1 or IP6K3

To verify that the IP6K1 and IP6K3 isoforms exhibit similar effects, but do not compensate for each other, we used specific shRNA oligonucleotides to reduce the levels of each isoform in rat hippocampal neurons in culture. Neurons were transfected at the time of plating with VGLUT1-pH or VGLUT2-pH and infected at DIV7 with lentiviruses expressing constructs containing either IP6K1- or IP6K3-specific shRNA hairpins, along with mTagBFP as a reporter to monitor infection efficiency. We quantitated the amount of knockdown in cells expressing blue fluorescent protein using immunocytochemistry with antibodies to IP6K1 and IP6K3. Knockdown of endogenous rat IP6K1 and 3 in hippocampal neurons is ∼50% for each construct (Supplementary Figure 2; Rao et al., 2014b; Fu et al., 2015). Lentivirus packaged with vector expressing only mTagBFP was used as a control. At DIV14, neurons were electrically stimulated at 10 Hz for 60 s (Figure 2D). In VGLUT2-pH expressing neurons, infection with vector containing shRNA against IP6K1 or IP6K3 accelerates the rate of VGLUT2-pH endocytosis (control, green, τ = 57.87 ± 7.91 s; IP6K1 KD, red stripes, τ = 24.48 ± 3.73 s, ^**^p < 0.01, ANOVA; IP6K3 KD, gray stripes, τ = 27.65 ± 2.68 s, ^**^p < 0.01, ANOVA), similar to the KOs. Thus, genetic deletion, knockdown, and pharmacological inhibition of IP6Ks accelerate VGLUT2-pH endocytosis.

### Exocytosis of VGLUT1- and 2-pH is increased by pharmacological inhibition of inositol hexakisphosphate kinases

**FIGURE 3:**
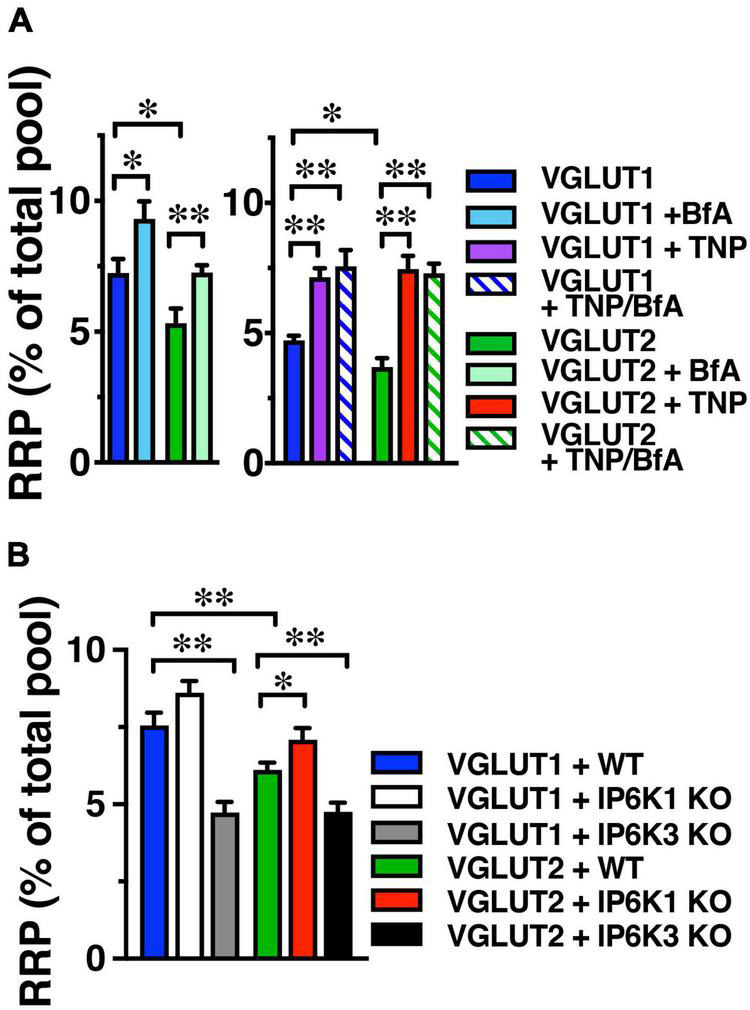
IP6K inhibition increases VGLUT-pHs in the RRP. **(A)** The fraction of VGLUT1 and 2-pH that undergoes exocytosis in response to a 100 Hz 20 AP stimulus to release the RRP is larger in the presence of BfA (left) and TNP (right). There is no additive effect of TNP and BfA. BfA increases the amount of VGLUT1- and 2-pH in the RRP to a lesser extent than TNP. Data are means ± SEM of ΔF/F_0_ normalized to total fluorescence over at least 35 boutons per coverslip from 9 to 12 coverslips per construct and at least three independent cultures for both experiments. **(B)** IP6K1 KO does not significantly increase VGLUT2-pH in the RRP, while IP6K3 KO decreases VGLUT1- and 2-pH in the RRP. Data are means ± SEM of ΔF/F_0_ normalized to total fluorescence over at least 40 boutons per coverslip from 6 to 12 coverslips per construct and at least three independent cultures. *p < 0.05, ^**^p < 0.01, ANOVA.

The product of IP6Ks, 5-IP7, has been demonstrated to inhibit exocytosis by a mechanism involving binding to a C2 domain of synaptotagmin (Irvine and Cullen, 1996; Lee et al., 2016). Knockdown of IP6K1 increased exocytosis induced by a short 10 Hz 10 s stimulus (Lee et al., 2016). IP6K1 KO decreases IP7 levels by 50–70% (Bhandari et al., 2008; Chakraborty et al., 2010). To measure the amount of VGLUT-pH released upon stimulation, we used alkaline trapping with bafilomycin, an inhibitor of the H^+^-ATPase. Bafilomycin added to the recording medium blocks reacidification of vesicles that have undergone exocytosis and taken up the drug, eliminating fluorescence changes due to the endocytic component of SV recycling, to reveal only exocytosis (Sankaranarayanan and Ryan, 2001). To measure VGLUT-pHs released in the readily releasable pool (RRP), we use a short 20 AP stimulus (100 Hz for 0.2 s) (Ariel and Ryan, 2010). The RRP is measured as a fraction of the total pool, which is revealed by addition of 50 μM NH_4_Cl, and normalized to the amount of VGLUT1-pH (blue) or VGLUT2-pH (green) in the DMSO control (Figure 3). We tested the effect of treating neurons with the pan-IP6K inhibitor TNP for 2 h (Padmanabhan et al., 2009), on VGLUT1- and 2-pH exocytosis. In contrast to the VGLUT isoform-specific endocytic response after prolonged stimulation, TNP increases the amount of VGLUT1-pH (purple, 7.16 ± 0.34% of total pool) compared to DMSO control (4.73 ± 0.17%, ^**^p < 0.01, ANOVA) in the RRP. TNP treatment also increases VGLUT2-pH (red, 7.46 ± 0.52%) in the RRP, compared to DMSO control (3.69 ± 0.35%, ^**^p < 0.01, ANOVA) (Figure 3A). We tested pretreatment with the clathrin adaptor AP-1 and AP-3 inhibitor brefeldin A (BfA) in this assay as well. Surprisingly, treatment with BfA exerts a similar effect, increasing the amount of both VGLUT1-pH (light blue, 9.31 ± 0.67%) released from the RRP compared to control (7.24 + 0.53%, *p < 0.05, ANOVA) and VGLUT2-pH (light green, 7.25 ± 0.29%), compared to DMSO control (5.32 ± 0.57%, ^**^p < 0.01, ANOVA). TNP appears to have a larger effect on RRP size than BfA, but BfA treatment has no additional effect on the enhancement of the RRP of either VGLUT1-pH (blue stripes, 7.57 ± 0.63%) or VGLUT2-pH (green stripes, 7.29 ± 0.38%), which could suggest that AP-1/3 adaptors and IP7 function in the same pathway, that the use-dependent BfA pretreatment is incomplete as not all SVs that respond to the RRP stimulus have undergone a round of endocytosis, or that the effect of IP7 is simply larger (Figure 3A). However, taken together, pharmacologic inhibition of AP-1/3 or IP6Ks increase both VGLUT1- and 2-pHs in the RRP.

We also tested the effect of IP6K KO on the release of VGLUT1- and 2-pH in the RRP. IP6K1 KO significantly increases the amount of VGLUT2-pH released from the RRP, here shown as a fraction of the total pool, revealed by addition of 50 μM NH_4_Cl (VGLUT2-pH in WT, green, 6.11 ± 0.23%; in IP6K1 KO, red, 7.08 ± 0.37%, *p < 0.05, ANOVA). IP6K1 KO increases the amount of VGLUT1-pH released from the RRP, but this difference is not significant (RRP size: VGLUT1-pH in WT, blue, 7.55 ± 0.43%; in IP6K1 KO, white, 8.66 ± 0.38%) (Figure 3B). IP6K3 KO, in contrast, results in a significant decrease in both VGLUT1- and 2-pH in the RRP, compared to WT (VGLUT1-pH in IP6K3 KO, gray, 4.73 ± 0.34%; VGLUT2-pH in IP6K3 KO, black, 4.76 ± 0.29%, ^**^p < 0.01, ANOVA) We note, however, that IP6K3 KO is associated with defects in synapse formation (Fu et al., 2015; Rojas et al., 2019), so changes in RRP size may reflect cytoskeletal abnormalities in synapse stabilization, for example (Featherstone et al., 2001; Pielage et al., 2005). Thus, the effect of IP6K1 KO on increasing VGLUT-pHs in the RRP more closely resembles IP6K pharmacologic inhibition, while IP6K3 KO demonstrates an opposite effect, but may reflect defects in axon pathfinding and synapse development.

### Spontaneous and miniature postsynaptic currents

**FIGURE 4:**
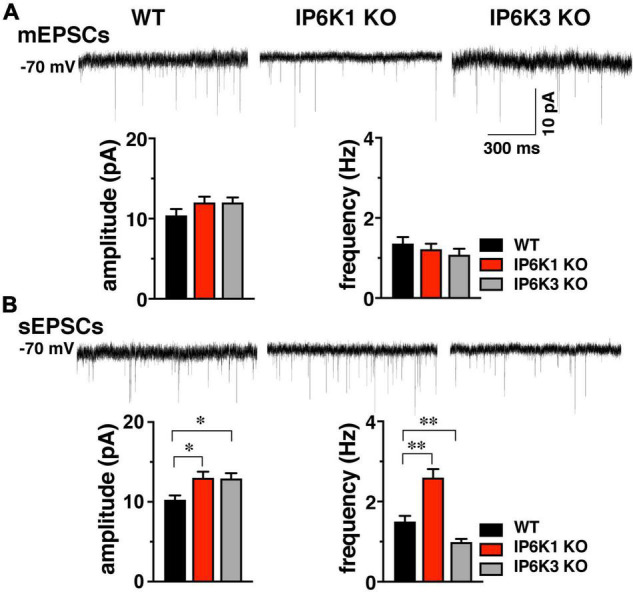
Effect of IP6K KOs on EPSCs in striatum, which contains both VGLUT1 and 2 synapses. **(A)** *Top:* representative mEPSC traces from WT (*n* = 10) and IP6K1 (*n* = 12) and IP6K3 (*n* = 8) KO mice under voltage clamp at –70 mV in the presence of 1 μM TTX. *Bottom:* averaged amplitudes and frequencies indicate no differences between genotypes. **(B)** *Top:* representative traces of spontaneous EPSCs. *Bottom:* average sEPSC amplitudes are larger in both IP6K1 (*n* = 9) and IP6K3 (*n* = 10) KO mice, compared to WT (*n* = 8). Frequency of sEPSCs is increased in IP6K1 KO and decreased in IP6K3 KO, compared to WT. *p < 0.05, ^**^p < 0.01, ANOVA.

To determine if basic synaptic characteristics are altered by deletion of IP6K1 or IP6K3, miniature (mEPSC) and spontaneous (sEPSC) excitatory postsynaptic currents from the striatum were measured (Figure 4). We took advantage of an angled slice preparation that preserves the thalamostriatal and corticostriatal fibers (Smeal et al., 2007), thus a single preparation can have both VGLUT2 and VGLUT1 inputs to striatum (Raju et al., 2006; Ding et al., 2008; Doig et al., 2010). Miniature EPSCs were analyzed in the presence of 1 μM tetrodotoxin (TTX) (Figure 4A). No significant differences in mEPSC amplitude were found between WT (10.40 ± 0.81 pA, *n* = 10) and IP6K1 KO (12.00 ± 0.74 pA, *n* = 12) or IP6K3 KO mice (12.00 ± 0.63 pA, *n* = 8). Similarly, there were no differences in mEPSC frequency between WT and IP6K KOs (WT: 1.36 ± 0.16 Hz, *n* = 10; IP6K1 KO: 1.22 ± 0.14 Hz, *n* = 12; IP6K3 KO: 1.08 ± 0.15 Hz, *n* = 8, ANOVA).

In comparison to mEPSCs, where synaptic events are limited to individual synapses and a single mini event can be considered a direct result of the release of a single vesicle in the absence of action potentials, sEPSCs can reflect action potential driven events due to the intrinsic properties of the presynaptic cell, and thus may provide information about general network drive and activity. Quantification of the amplitude and frequency of sEPSCs shows that mean amplitudes are increased in both IP6K1 KO (13.00 ± 0.78 pA, *n* = 9) and IP6K3 KO mice (12.93 ± 0.67 pA, *n* = 10), compared to WT [10.25 ± 0.55 pA, *n* = 8, *p<0.05 for both, ANOVA (Figure 4B)]. IP6K1 KO mice exhibit significantly higher sEPSC frequencies (2.60 ± 0.21 Hz, *n* = 9) compared to WT (1.50 ± 0.14 Hz, *n* = 8, ^**^p<0.01, ANOVA). However, frequencies of sEPSCs are decreased in IP6K3 KO (0.99 ± 0.08 Hz, *n* = 10, ^**^p<0.01, ANOVA), perhaps consistent with impairments in synapse formation (Fu et al., 2015). Thus, IP6K KOs may exhibit altered network activity.

### Short-term plasticity in IP6K1 and IP6K3 knockout mice

**FIGURE 5:**
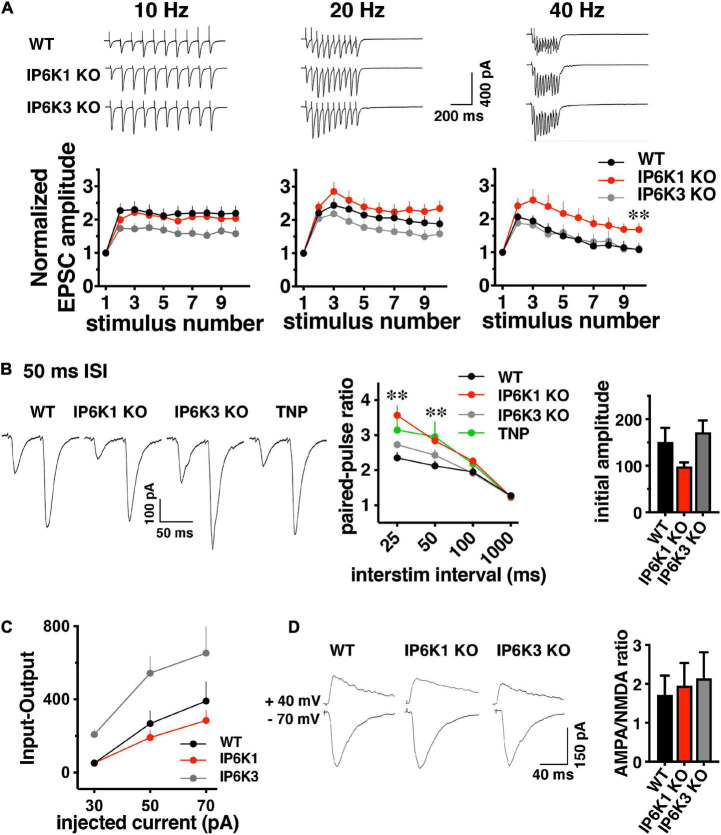
Short-term facilitation in predominantly VGLUT1 expressing Schaffer collateral synapses. **(A)** *Top:* representative traces of EPSC trains evoked at 10, 20, and 40 Hz stimulation recorded at –70 mV in whole cell configuration. *Bottom:* normalized EPSC amplitudes show activity dependent facilitation of IP6K1 KO compared to WT at 40 Hz (*p < 0.05 and **p < 0.01). Data are means ± SEM from *n* = 9–11 cells. **(B)** *Left:* representative examples of postsynaptic responses induced by pairs of depolarization-evoked action potentials (50 ms apart) in the presynaptic cell. Stimulus artifacts are blanked. *Middle:* summary plot of paired pulse ratio (PPR) calculated at 25, 50, 100, and 1000 ms ISI in the same cells, showing higher facilitation at shorter ISIs. Deletion of IP6K1 (red) increases paired pulse facilitation, compared to WT (black, *p < 0.05 and **p < 0.01). Treatment with the IP6K inhibitor TNP is similar to IP6K1 KO (*n* = 8–11, *p* > 0.05). *Right*: initial EPSC amplitudes in paired-pulse. **(C)** Input-output curve demonstrating EPSC amplitudes at different current intensities (*n* = 4–8). **(D)** *Left:* representative traces of AMPA and NMDA receptor mediated EPSCs. *Right:* quantitation indicates no significant differences in AMPA/NMDA ratio between WT and IP6K KO mice (*n* = 5–6).

Since we observed effects of IP6Ks on the release of both VGLUT-pH isoforms in the RRP, we first investigated the early phase of neurotransmitter release. We characterized short-term plasticity in IP6K1 and IP6K3 KO mice in hippocampal Schaffer collateral synapses, where glutamate release is mainly dependent on VGLUT1 (Fremeau et al., 2004a). Synaptic function in response to stimulus trains of ten action potentials was examined at different frequencies: 10, 20, and 40 Hz in acute hippocampal slices. WT synapses exhibit synaptic facilitation that is larger at higher frequencies and declines gradually as stimulation progresses (Figures 5A,B). IP6K1 KO synapses exhibit higher levels of synaptic facilitation at 40 Hz (facilitation at stimulus 10: 1.68 ± 0.20, *n* = 10) compared to WT (1.08 ± 0.15, *n* = 9, ^**^p < 0.01, ANOVA), whereas at 10 Hz the IP6K1 KO exhibits similar facilitation (2.04 ± 0.12, *n* = 11) compared to WT (2.19 ± 0.21, *n* = 9). At 40 Hz, IP6K3 KO synapses exhibit similar levels of synaptic facilitation (1.1 ± 0.65, *n* = 11) compared to WT. IP6K3 KO synapses trend toward lower levels of facilitation than WT at 10 Hz (1.58 ± 0.21, *n* = 11, *p* = 0.051) but this difference was not significant.

These results are in sharp contrast to the phenotypes of a reported CRISPR generated IP6K1 KO, which exhibited a lower initial PPR than WT and decreased release at high frequencies (Park et al., 2020). We thus compared paired-pulse ratio (PPR) between WT, IP6K KOs, and treatment with the pharmacological IP6K inhibitor, TNP. Four different interstimulus intervals (ISIs) were compared: 25, 50, 100 and 1000 ms (Figure 5B). Genetic deletion of IP6K1 results in an increase in activity dependent synaptic facilitation and initial PPR compared to WT at a 50 ms interstimulus interval (ISI) (PPR_50 ms_ WT: 2.13 ± 0.13, *n* = 8, IP6K1 KO: 2.84 ± 0.18, *n* = 11, ^**^p < 0.01, ANOVA). Pharmacologic inhibition of IP6Ks by TNP also results in synaptic facilitation, though this does not reach significance (PPR_50 ms_: 2.96 ± 0.4, *n* = 8, *p* = 0.07). IP6K3 KO results in increased facilitation compared to WT, which was not significant (PPR_50 ms_: 2.4 ± 0.14, *n* = 11, *p* = 0.15) and to a lesser extent than IP6K1 or TNP.

These effects may be driven by differences in initial current amplitudes in paired pulse recordings, although these do not reach significance [WT, 151.0 ± 30.53 pA; IP6K1 KO, 98.49 ± 8.92 pA; IP6K3 KO, 171.7 ± 25.67 pA (Figure 5B, right panel)]. Input-output responses to a single stimulus at different intensities show similar current amplitudes in IP6K1 KO compared to WT [30 pA injected current: WT (*n* = 8), 51.12 ± 12.75 pA; IP6K1 KO (*n* = 8), 52.34 ± 12.28 pA, IP6K3 KO (*n* = 4), 208.3 ± 32.91 pA; 50 pA injected: WT, 268.5 ± 69.80 pA; IP6K1 KO, 191.2 ± 39.12 pA; IP6K3 KO: 544.2 ± 91.85 pA; 70 pA injected: WT, 390.6 ± 106.1 pA; IP6K1 KO, 284.6 ± 55.18 pA, IP6K3 KO, 653.2 ± 142.9 pA (Figure 5C)].

To determine whether these effects might be due to postsynaptic changes, we measured the ratio of α-amino-3-hydroxy-5-methyl-4-isoxazolepropionic acid (AMPA) and N-methyl-D-aspartate (NMDA) receptor mediated currents in the KOs by recording EPSCs at −70 mV and +40 mV (Figure 5D). There is no significant difference in the AMPA/NMDA ratio between WT (1.95 ± 0.49, *n* = 6) and IP6K1 KO (1.95 ± 0.58, *n* = 6) or IP6K3 KO (2.14 ± 0.66, *n* = 5, *p* > 0.05, ANOVA), suggesting the effects of IP6K KOs on synaptic facilitation are presynaptic in origin. Taken together, the data indicate IP6K1 KO increases synaptic facilitation.

### Response to repetitive stimulation in VGLUT1 and VGLUT2 pathways

**FIGURE 6:**
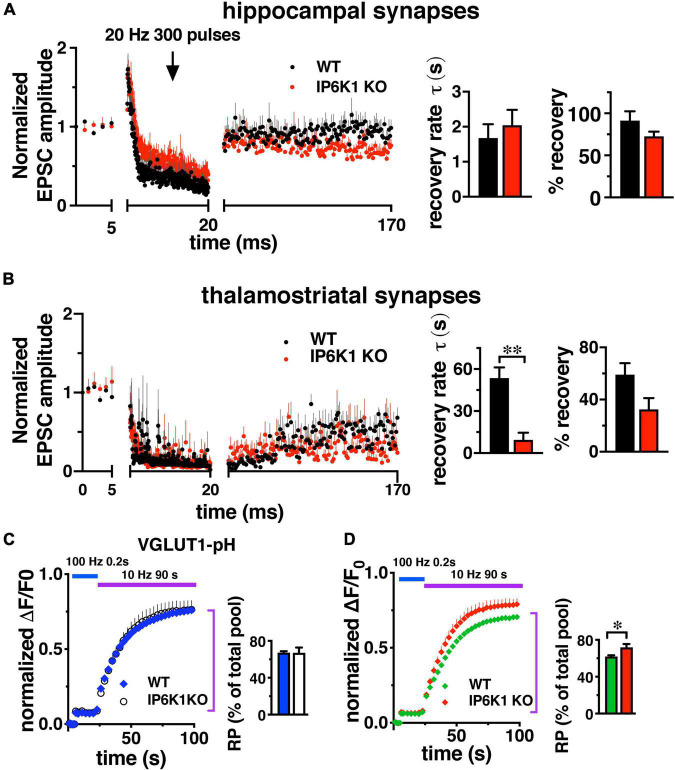
Response of VGLUT1 expressing hippocampal synapses and VGLUT2 expressing thalamostriatal synapses to prolonged stimulation. **(A)** CA1: baseline EPSCs evoked at 1 Hz and followed by a 20 Hz 300 pulse train in WT (*n* = 6) and IP6K1 KO (*n* = 5). Recovery from short-term depression was measured at 1 Hz for 150 s. Currents were normalized to the average amplitudes of baseline EPSCs. Recovery rate time constant was approximated with a single exponential fit. Percentage recovery was calculated based on the recovered EPSC amplitudes. **(B)** Thalamostriatal: same as in **A** for WT (*n* = 5) and IP6K (*n* = 6). ^**^p < 0.01, *t*-test. All data are presented as means ± SEM unless otherwise noted. **(C,D)** Fluorescence changes representing the amount of VGLUT2-pH, but not VGLUT1-pH, released from the SV recycling pool (RP, purple bar) after release of the RRP (blue bar) is increased in IP6K1 KO mice, compared to control (*p < 0.05). Data are means ± SEM of ΔF/F_0_ normalized to total fluorescence over at least 30 boutons per coverslip from 6 to 9 coverslips and three independent cultures.

In contrast to short stimuli, we observed VGLUT isoform-specific differences in endocytosis in IP6K KOs after intense stimulation by live-cell imaging in cultured neurons (Figures 2B,C). To determine the effect of prolonged stimulation in VGLUT1 and VGLUT2 pathways, we compared the response of Schaffer collateral synapses, which mainly express VGLUT1 and exhibit synaptic facilitation, and thalamostriatal synapses, which mainly express VGLUT2 and exhibit synaptic depression (Raju et al., 2006; Smeal et al., 2007; Ding et al., 2008; Doig et al., 2010; Figures 6A,B). However, under high intensity or prolonged stimulation conditions, some hippocampal synaptic vesicles may also recycle by AP1/3-dependent, bulk endocytosis (Voglmaier et al., 2006; Cheung and Cousin, 2012). After 1 Hz baseline stimulation, 300 pulses were applied at 20 Hz (15 s), after which synapses recovered under 1 Hz stimulation. Responses were normalized to baseline amplitudes. During the 150 s 1 Hz recovery period in VGLUT1-containing hippocampal synapses (Figure 6A), WT EPSCs recover to a level of 91.30 ± 11.02% (black, *n* = 6) and IP6K1 KO recovers to a level of 72.61 ± 5.69% (red, *n* = 5). Recovery in VGLUT2-containing thalamostriatal synapses is less complete, reaching a level of only 59.18 ± 8.76% in the WT (*n* = 5) and 32.52 ± 8.64% in the IP6K1 KO (*n* = 6). The rate of recovery in hippocampal synapses is modeled here as a single exponential and does not differ between WT (τ = 1.69 ± 0.39 s) and IP6K1 KO (τ = 2.04 ± 0.44 s). The recovery is slower and less complete in thalamostriatal synapses (Figure 6B), however, the IP6K1 KO synapses recover significantly faster (τ = 9.38 ± 5.17 s), than WT (τ = 53.56 ± 7.59 s, ^**^p < 0.01). Thus, consistent with VGLUT isoform specific effects on endocytosis after intense stimulation, IP6K1 KO speeds recovery of neurotransmitter release in predominantly VGLUT2 synapses, while there is no effect on VGLUT1 synapses.

To measure how IP6K1 KO affects the amount of transporter that undergoes exocytosis under prolonged stimulation, alkaline trapping was again used in VGLUT-pH transfected neurons. The RRP is first released with a 20 AP stimulus at 100 Hz, then a 900AP stimulus (10 Hz 90 s) is applied to release the entire recycling pool (RP, Figure 6C). Subsequent addition of 50 mM NH_4_Cl reveals the total pool (Fernandez-Alfonso and Ryan, 2008; Foss et al., 2013). The proportion of VGLUT1-pH that undergoes exocytosis from WT neurons with this 900 AP stimulus (blue, 67.28 ± 1.57% of total pool) is similar in IP6K1 KO neurons (white, 67.11 ± 5.80%) (Figure 6C). The amount of VGLUT2-pH in the RP in IP6K1 KO cells (red, 71.86 ± 3.65%) is significantly higher compared to WT (green, 62.09 ± 1.17%, *p < 0.05) (Figure 6D). Thus while IP6Ks exert similar effects on both VGLUT1 and VGLUT2 isoforms at early time points, IP6K1 affects the amount of VGLUT2-pH released, but not VGLUT1-pH, in response to a 900 AP stimulus that releases the remainder of the recycling pool.

### Response to repeated action potential trains

**FIGURE 7:**
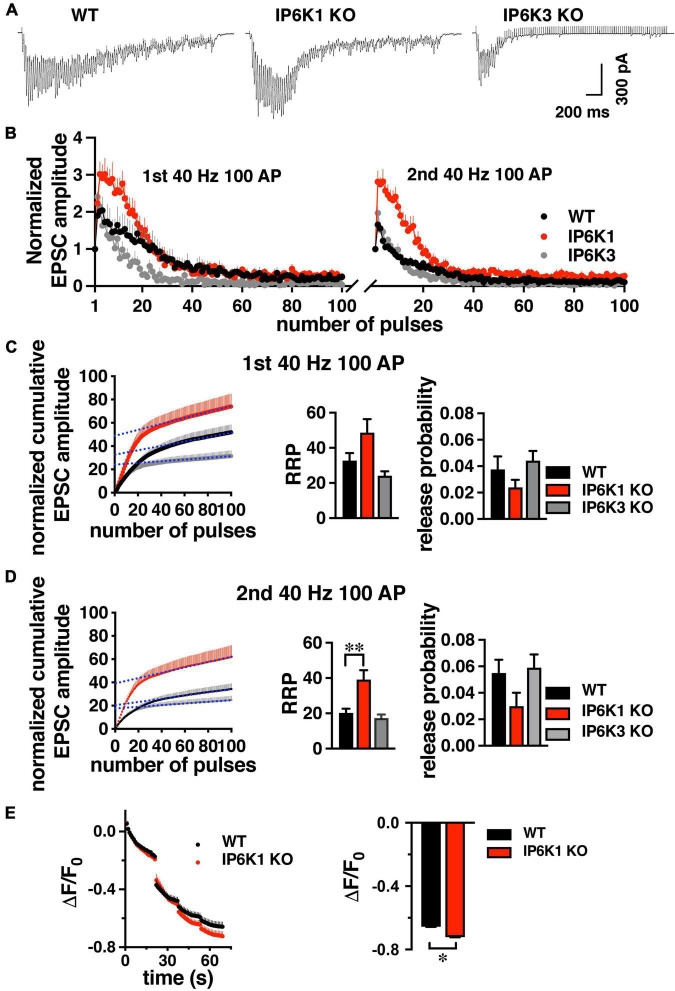
Response of WT and IP6K1 KO in hippocampal VGLUT1 synapses to high frequency repeated stimulation. **(A)** Representative traces of CA1 EPSCs evoked by 100 stimuli at 40 Hz in WT (*n* = 7) and IP6K1 (*n* = 6) and IP6K3 (*n* = 5) KO slices. **(B)** Summary plot of EPSC amplitude, normalized to the first EPSC amplitude, in response to two 40 Hz 100 pulse stimuli with a 7 min recovery between stimulus trains. **(C)** Cumulative EPSC amplitude during the first 40 Hz stimulus train in WT and IP6K1 KO. EPSC amplitudes are normalized to the first stimulus. The last 20 data points are fitted to a linear regression and extrapolated to time zero to estimate RRP size. Release probability is estimated by dividing the first EPSC amplitude by the RRP size. **(D)** Cumulative EPSC amplitude as in **C** for the second 40 Hz stimulus train. ^**^p < 0.01, ANOVA. **(E)** Cultured hippocampal WT and IP6K1 KO neurons were loaded with FM1-43 using an 80 Hz 10 s stimulus. After washing, the extent of dye release is measured upon unloading from the RRP with a 30 Hz 2 s stimulus, then the remaining recycling pool with three 40 Hz 10 s stimuli. All data are presented as means ± SEM, unless otherwise noted. *p < 0.05, *t*-test.

Under intense stimulation conditions, VGLUT1-expressing cerebellar and hippocampal cells in culture can also undergo recycling by an AP-1/3 and GSK-3-dependent endocytosis pathway. We therefore tested IP6K KOs in a repeated stimulation paradigm that allows use of the more robust hippocampal CA1 pathway (Clayton et al., 2008; Cheung and Cousin, 2012; Bonnycastle et al., 2022). GSK-3 phosphorylates dynamin, responsible for pinching off SVs (Cousin and Robinson, 2001), upon a second stimulus (Clayton et al., 2010). CA1 neurons were challenged with high frequency prolonged stimulation (40 Hz 100 AP). After a 7 min of recovery period, the response to a second 40 Hz 100 AP stimulus was measured (Figures 7A,B).

Estimation of RRP size from normalized cumulative EPSC amplitudes by back-extrapolation to time 0 (*y*-intercept), indicates a larger RRP for IP6K1 KO in the second stimulation (first stimulation: 48.67 ± 7.72, *n* = 6; second stimulation: 39.17 ± 5.26, *n* = 6), in comparison with WT (first stimulation: 32.71 ± 4.37, *n* = 7, *p* = 0.09; second stimulation: 20.29 ± 2.40, *n* = 7, ^**^p < 0.01) (Figures 7C,D). In contrast to IP6K1, IP6K3 KO demonstrates a decreased RRP in the second stimulation (first stimulation: 24.20 ± 2.46, *n* = 5; second stimulation: 17.40 ± 1.94, *n* = 5, *p < 0.05, ANOVA). These results are broadly consistent with RRP comparisons obtained from live-cell imaging experiments (Figure 3B). We also calculated initial release probability (P_*r*_) in the CA1 synapses, related to RRP size (Figures 7C,D; Schneggenburger et al., 1999; Thanawala and Regehr, 2013). We note that after the first high frequency stimulation, P_*r*_ is increased in all groups measured, but the changes are not significant (first stimulation: WT, 0.037 ± 0.01, *n* = 7; IP6K1 KO, 0.024 ± 0.01, *n* = 6; IP6K3 KO, 0.044 ± 0.01, *n* = 5; second stimulation: WT, 0.055 ± 0.01; IP6K1 KO, 0.032 ± 0.01; IP6K3 KO, 0.059 ± 0.01). Thus, the difference in RRP between WT and IP6K1 is larger upon repeated stimulation, with a greater shift toward release earlier in the stimulus train.

Finally, we used a high intensity 80 Hz stimulus, shown to induce both CME and ADBE in predominantly VGLUT1-expressing hippocampal cultures to examine how IP6K1 KO affects recycling of SVs (Wenzel et al., 2012). Here we use the styryl dye FM1-43 to label SV membranes rather than SV cargo. Neurons are loaded with FM1-43 with an 80 Hz 10 s stimulus, excess dye washed out, and then unloaded with a sequential unloading strategy to first release the RRP with a 30 Hz 2 s stimulus, then three 40 Hz 10 s action potential trains to release the recycling pool (Figure 7E; Cheung and Cousin, 2012). A larger decrease in fluorescence from IP6K1 KO synapses (ΔF/F_0_ = −0.72 ± 0.01, *n* = 5), compared to WT (ΔF/F_0_ = 0.67 ± 0.01, *n* = 7, *p < 0.05 *t*-test), confirms more SVs are released from the IP6K1 KO recycling pool.

## Discussion

Higher inositol polyphosphates (IP4-7) have been shown to interact with several proteins important for endocytosis and synaptic vesicle recycling including the clathrin adaptor AP-2 (Voglmaier et al., 1992), AP180 (Norris et al., 1995; Ye et al., 1995) and the C2B domain of synaptotagmin (Llinas et al., 1994; Schiavo et al., 1995; Lee et al., 2016). More recently it was shown that 5-IP7 has a 45-fold higher affinity for synaptotagmin than IP6 and negatively regulates SV vesicle fusion. Knockdown of IP6K1 increased SV exocytosis (Lee et al., 2016). While a CRISPR knockout of IP6K1 shows increased initial release probability compared to WT, it exhibits decreased facilitation and decreased SV exocytosis upon high frequency stimulation (Park et al., 2020). In the case of insulin granules, IP6 and IP7 produced by IP6K1 promote insulin secretion by vesicle fusion (Illies et al., 2007; Barker et al., 2009). IP6K1 is upstream of proteins particularly important for SV recycling by bulk endocytosis, GSK-3 and AP-3 (Clayton et al., 2010; Cheung and Cousin, 2012). GSK-3 phosphorylation of dynamin under repeated or intense stimulation is associated with SV reformation from bulk endosomes (Clayton et al., 2010). IP6K1 has been shown to activate GSK-3 by several mechanisms–*via* direct activation by the IP6K1 protein, activation by its product 5-IP7, and indirectly by 5-IP7-mediated inhibition of the GSK-3 negative regulator Akt (Chakraborty et al., 2014). IP7 also pyrophosphorylates the multimeric clathrin adaptor AP-3 (Saiardi et al., 2004; Azevedo et al., 2009). We previously demonstrated that AP-3 is important for recycling of VGLUT2 (Li et al., 2017), and may also be involved in recycling of VGLUT1 under prolonged or intense stimulation conditions (Voglmaier et al., 2006; Cheung and Cousin, 2012; Wenzel et al., 2012). We therefore tested the effects of IP6K inhibition on VGLUT1 and 2 exo- and endocytosis under moderate and intense stimulation conditions, early and late in the stimulus.

We demonstrate that both IP6K1 and IP6K3 isoforms are at least partly localized to axons, suggesting both isoforms might play a role in SV recycling. However, IP6K3 has been shown to localize extensively to dendrites and act postsynaptically (Fu et al., 2015). Under modest stimulation conditions (10 Hz 1 min), pharmacologic inhibition and knockout of IP6K1 or IP6K3 speeds endocytosis of VGLUT2-pH, which utilizes the AP-3 adaptor protein, with no significant effect on VGLUT1-pH, which relies mainly on AP-2 under these conditions. Importantly, acute knockdown of IP6Ks showed a similar effect as genetic deletion. In both KO and knockdown, IP6K1 and IP6K3 have a similar effect on endocytosis, but do not compensate for each other. It may be that the mechanisms mediating effects observed here are exquisitely sensitive to small changes in the IP6/7 ratio (Menniti et al., 1993; Lee et al., 2016; Chakraborty, 2018). The IP6K isoforms may also be localized differently and exert their effects locally either by local production of IP7 or a direct effect of the IP6K on an effector. Pyrophosphorylation by IP6K is non-enzymatic, so the pyrophosphate moiety may be transferred directly from IP6K to its target, analogous to ubiquitination, thus differential subcellular localization may result in differences in function as well (Bhandari et al., 2007; Azevedo et al., 2009; Werner et al., 2010). It is also worth noting that the high concentrations of cellular IP6 and presumably IP7 would likely be insoluble in physiological levels of Mg^2+^, however, IP6 remains NMR visible and therefore soluble, so some physical or functional compartmentalization has been proposed (Hanley et al., 1988; Irvine et al., 1988; Carpenter et al., 1989; Poyner et al., 1993).

Using a short stimulus to release the RRP, we show that pharmacologic inhibition of IP6Ks increases exocytosis, in this case of both VGLUT1- and VGLUT2-pH. The AP-1/3 inhibitor BfA also increases exocytosis of both VGLUT1- and VGLUT2-pH, though to a lesser extent than TNP and has no additive effect. It is interesting that an inhibitor of endocytic adaptors affects exocytosis. This may still occur through an endocytic process as BfA is a use-dependent inhibitor, so it may be that only SVs that underwent a round of exocytosis would be affected. This may also lead to an incomplete effect if some different SVs are accessed upon the test stimulus. It may also be due to an endocytic process during the stimulus affecting release site clearance for example (Neher, 2010; Hua et al., 2013). Ultrafast endocytosis is a form of bulk endocytosis, so may use shared mechanisms and its time scale suggests it could be a rate limiting step for SV exocytosis (Watanabe et al., 2013a,b; Watanabe and Boucrot, 2017).

Isoform-specific knockout, however, does show different effects of IP6K1 and IP6K3 on early exocytosis (Figure 3B). IP6K1 KO increases VGLUT1- and -2-pHs in the RRP, while IP6K3 KO decreases release. Thus, while IP6K1 and IP6K3 KOs exert similar effects on endocytosis after stimulation, the isoforms show opposite effects on exocytosis earlier in the stimulus. IP6K1 and IP6K3 KO also exert different effects on the early phase of glutamate release (Figure 5). In paired pulse experiments, both strains show increased PPR but IP6K1 KO more closely resembles TNP than IP6K3 KO (Figure 5B). IP6K1 KO increases facilitation compared to WT upon high frequency stimulation (Figure 5A). During prolonged 20 Hz stimulation (Figure 6A) IP6K1 KO CA1 synapses appear to facilitate until the end of the stimulus, although the differences with WT do not reach significance. Whereas 40 Hz prolonged stimulation induces increased facilitation only during the first 20 stimuli in IP6K1 KO (Figures 7A,B). In contrast to IP6K1 KO, IP6K3 KO reduces facilitation compared to WT upon repeated stimulation (Figures 5A, 7A,B). However, it must be noted that IP6K3 KO mice have a reduced number of dendritic spines and subtle defects in synapse formation, which may interfere with interpretation of electrophysiological data recorded postsynaptically (Fu et al., 2015). Reduced spine number decreases the number of functional excitatory synapses and influences receptor density. These changes affect synaptic plasticity; spine loss is related to long-term depression (Zhou et al., 2004). Based on these results, IP6K3 mice may present an interesting line to investigate LTP/LTD in CA1 pyramidal neurons. On the other hand, IP6K1 KO has been shown to increase β-catenin levels which could stabilize synapses (Chakraborty et al., 2014).

We investigated the recovery of glutamate release after prolonged 20 Hz stimulation to deplete the recycling pool of SVs. We compared synapses where glutamate packaging is largely dependent on VGLUT1 and exhibit paired pulse facilitation (hippocampal CA1) versus synapses that depend on VGLUT2 and exhibit paired pulse depression (thalamostriatal). While the kinetics of recovery in CA1 appear similar between WT and IP6K1 KO, in thalamostriatal slices, the recovery of IP6K1 KO is faster than WT (albeit incomplete in both preparations) (Figure 6). These experiments may not have the time resolution to detect a faster second exponential of recovery, however (Dittman and Regehr, 1998). In the later phase of SV exocytosis, IP6K1 KO specifically increases the extent of VGLUT2-pH release from the recycling pool, but has no significant effect on VGLUT1-pH. However, under conditions that induce recycling of VGLUT1 by ADBE, repeated high frequency stimulus trains, IP6K1 KO results in a significantly different amount of glutamate release from the RRP compared to WT. In FM dye experiments, IP6K1 KO synapses also release more SVs from the recycling pool (Figure 7).

Taken together, the data presented here show that IP6Ks regulate both exocytosis and endocytosis, particularly under conditions that induce recycling by molecular machinery associated with bulk endocytosis. The effects of IP6K1 on exocytosis observed early in the stimulus period are consistent with the reported inhibition of exocytosis by 5-IP7 by a synaptotagmin-dependent mechanism (Lee et al., 2016). However, the effects on SV endocytosis and glutamate release ascertained in this work are different from a report using a CRISPR generated knockout mouse (Park et al., 2020). In contrast to the enhancement of endocytosis and glutamate release in the IP6K1 KO observed here, the CRISPR KO mouse exhibits less facilitation, markedly reduced current amplitudes, and impaired endocytosis compared to WT. While some phenotypes are rescued by exogenous expression of IP6K1 in both studies, it remains possible there are off-target effects of CRISPR responsible for some differences. It is also possible that there are strain differences or some unknown compensatory mechanisms in one of the KO mouse lines. We note, however, that pharmacological inhibition by TNP replicates effects of IP6K1 on paired pulse ratio and endocytosis in the present study. Additionally, relatively acute knockdown of IP6Ks in cell culture replicates the enhancement of endocytosis rate observed in the IP6K KOs.

The specific effects of IP6K1 KO on endocytosis under intense stimulation conditions and the known interactions of IP6K1 and IP7 with proteins important in ADBE suggest IP6K1 may act in this pathway, but further work would be needed to establish a link. However, there are some differences, particularly effects on exocytosis. IP6Ks might also affect the molecular machinery underlying a clathrin and AP-2 independent pathway (CIE), responsible for endocytosis of a subset of SV cargos under moderate, non-repeated stimulation conditions (Lopez-Hernandez et al., 2022). SV recycling and maintenance of neurotransmitter in SV pools are energy intensive processes (Rangaraju et al., 2014; Pulido and Ryan, 2021). Intense stimulation imparts considerable demands on cellular energy stores which in turn induces compensatory mechanisms which could also include AP-1/3 mediated trafficking (Gillingham et al., 1999; Ashrafi et al., 2017; Mukherjee et al., 2020). ATP levels are efficiently maintained in the presynapse in response to SV recycling by AMP kinase stimulated trafficking of the glucose transporter GLUT4 in a pathway parallel to SV recycling (Ashrafi et al., 2017). IP7 levels are also an order of magnitude lower than ATP levels and decreases in IP7 concentration would not be expected to result in changes in endocytosis rates due to any energy constraints. It is also surprising that relatively small changes in IP7 levels can have large effects. However, the involvement of inositol pyrophosphates and IP6Ks in cellular energy dynamics suggests IP7 could be an ideal signal to translate changes in neuronal firing into changes in trafficking. The specific regulation of individual SV protein recycling pathways by IP6K confers the potential for regulation of specific SV pools and thus neurotransmitter release from different neuronal pathways (Voglmaier and Edwards, 2007).

## Significance statement

SV recycling is required to maintain high rates of neuronal firing, but regulation of SV exocytosis and endocytosis also offers an opportunity to shape the pattern of neurotransmitter signaling. Different SV recycling pathways induced by different stimulation intensities and with different molecular machinery, recycling rates, and destinations can independently sort SV protein cargos. The vesicular transporters responsible for packaging glutamate, VGLUT1 and 2, are expressed in separate cortical and subcortical pathways and are sorted by different recycling machinery. With moderate stimulation IP6Ks specifically affect VGLUT2 endocytosis and could therefore differentially modulate cortical and subcortical glutamatergic circuits. The endocytosis pathways differentially depend on neuronal firing rate and IP6Ks also affect exocytosis, thus regulation of IP6Ks could dampen excess activity while allowing normal physiological transmission to proceed. At the intersection of energy metabolism and SV recycling, IP6Ks and their products, inositol pyrophosphates, are uniquely poised to signal changes in neuronal firing and cellular energy demand. Differential effects on individual SV protein sorting confers the potential for changes in the composition of SVs and SV pools with different release probabilities. Upstream modulators of SV protein recycling offer an opportunity to target presynaptic signaling mechanisms as potential therapeutic targets in neuropsychiatric disease.

## Data availability statement

The raw data supporting the conclusions of this article will be made available by the authors, without undue reservation.

## Ethics statement

The animal study was reviewed and approved by the University of California IACUC.

## Author contributions

HL, MD, and RR designed and conducted the experiments, contributed to the data analysis, and the preparation of the manuscript. SV contributed to conceptualization of the study, to data analyses and interpretation, and to preparation of the manuscript. All authors reviewed and approved the final version of the manuscript.

## Conflict of interest

The authors declare that the research was conducted in the absence of any commercial or financial relationships that could be construed as a potential conflict of interest.

## Publisher’s note

All claims expressed in this article are solely those of the authors and do not necessarily represent those of their affiliated organizations, or those of the publisher, the editors and the reviewers. Any product that may be evaluated in this article, or claim that may be made by its manufacturer, is not guaranteed or endorsed by the publisher.

## Supplementary material

SUPPLEMENTARY FIGURE 1 Antibodies against human IP6K1 and 2 recognize rat *myc-*IP6K proteins expressed in HEK293 cells. Available commercial antibodies were prepared against human IP6Ks. To verify reactivity of these antibodies against rat IP6Ks, rat IP6K cDNAs were subcloned into a pCMV vector with a *myc* tag inserted, and transfected into HEK293 cells. Proteins were double stained with rabbit anti-human IP6K1 (Genetex), or goat anti-human IP6K2 (Santa Cruz), and mouse anti-*myc* (Covance), and mouse FITC, or rabbit or goat Cy5 (Jackson). The *myc* antibodies colabel with IP6K1 (top) or IP6K2 antibodies (bottom).

SUPPLEMENTARY FIGURE 2 Representative primary neurons showing knockdown of rat IP6K1. Primary rat hippocampal neurons were infected with lentivirus containing either FHUGW vector or shRNA against rat IP6K1. Cells were probed for IP6K1 immunoreactivity. FHUGW vector contains blue fluorescent protein (BFP) as a marker. Knockdown of endogenous rat IP6K1 in hippocampal neurons is ∼50%.

SUPPLEMENTARY FIGURE 3 Knockdown of overexpressed *myc-*IP6K3. To verify knockdown of rat IP6K3 protein with a specific shRNA, *myc*-tagged rat IP6K3 proteins were first overexpressed in HEK 293 cells, then infected with either FHUGW vector control or shRNA against rat IP6K3. Fluorescence intensity of the immunoreactivity (FITC) of *myc*-tagged rat IP6K3 was compared with vector control. Vector treated cells show co-expression of BFP and *myc*-tagged protein, IP6K3 shRNA infected cells show reduced FITC (*myc*-tagged rat IP6K3) immunoreactivity. Knockdown of overexpressed rat IP6K3 is ∼35%.
