# Supplementary figures and images for: Inositol hexakisphosphate kinases differentially regulate trafficking of vesicular glutamate transporters 1 and 2
Source: Front Cell Neurosci. 2022 Jul 22;16:926794. doi: 10.3389/fncel.2022.926794 (PMC9355605; doi:10.3389/fncel.2022.926794)

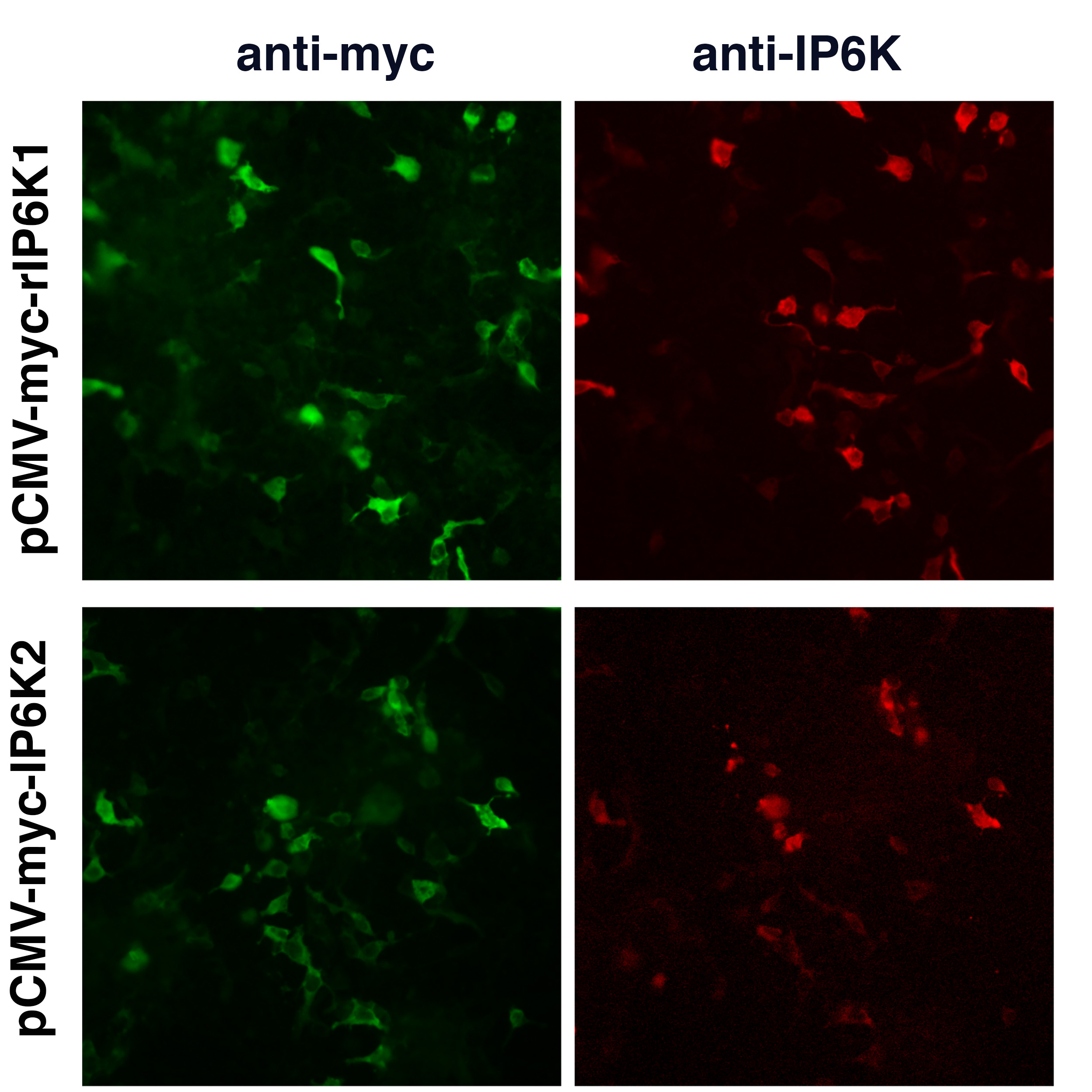

Supplement: SUPPLEMENTARY FIGURE 1 — Antibodies against human IP6K1 and 2 recognize rat myc-IP6K proteins expressed in HEK293 cells. Available commercial antibodies were prepared against human IP6Ks. To verify reactivity of these antibodies against rat IP6Ks, rat IP6K cDNAs were subcloned into a pCMV vector with a myc tag inserted, and transfected into HEK293 cells. Proteins were double stained with rabbit anti-human IP6K1 (Genetex), or goat anti-human IP6K2 (Santa Cruz), and mouse anti-myc (Covance), and mouse FITC, or rabbit or goat Cy5 (Jackson). The myc antibodies colabel with IP6K1 (top) or IP6K2 antibodies (bottom). [file Image_1.JPEG]

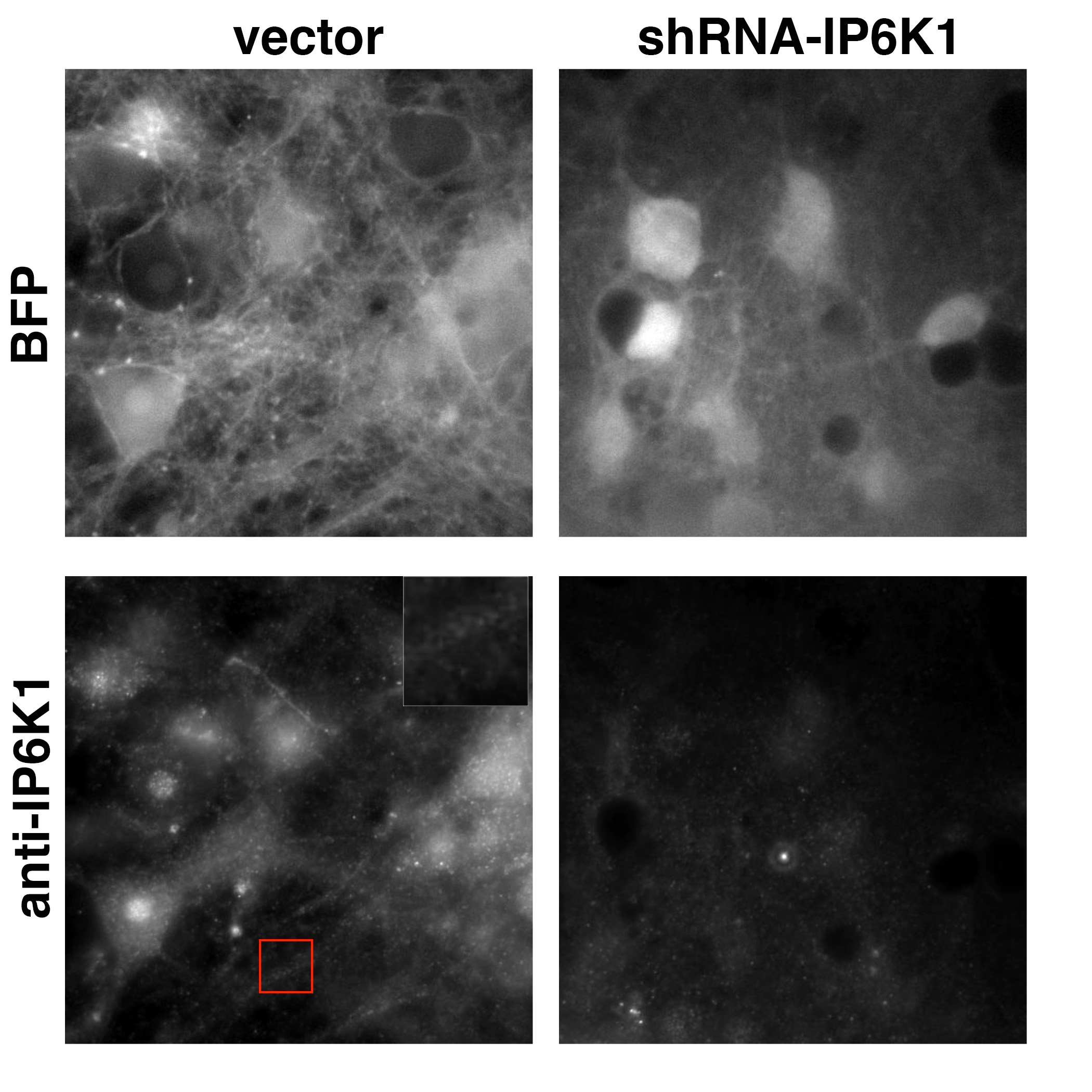

Supplement: SUPPLEMENTARY FIGURE 2 — Representative primary neurons showing knockdown of rat IP6K1. Primary rat hippocampal neurons were infected with lentivirus containing either FHUGW vector or shRNA against rat IP6K1. Cells were probed for IP6K1 immunoreactivity. FHUGW vector contains blue fluorescent protein (BFP) as a marker. Knockdown of endogenous rat IP6K1 in hippocampal neurons is ∼50%. [file Image_2.JPEG]

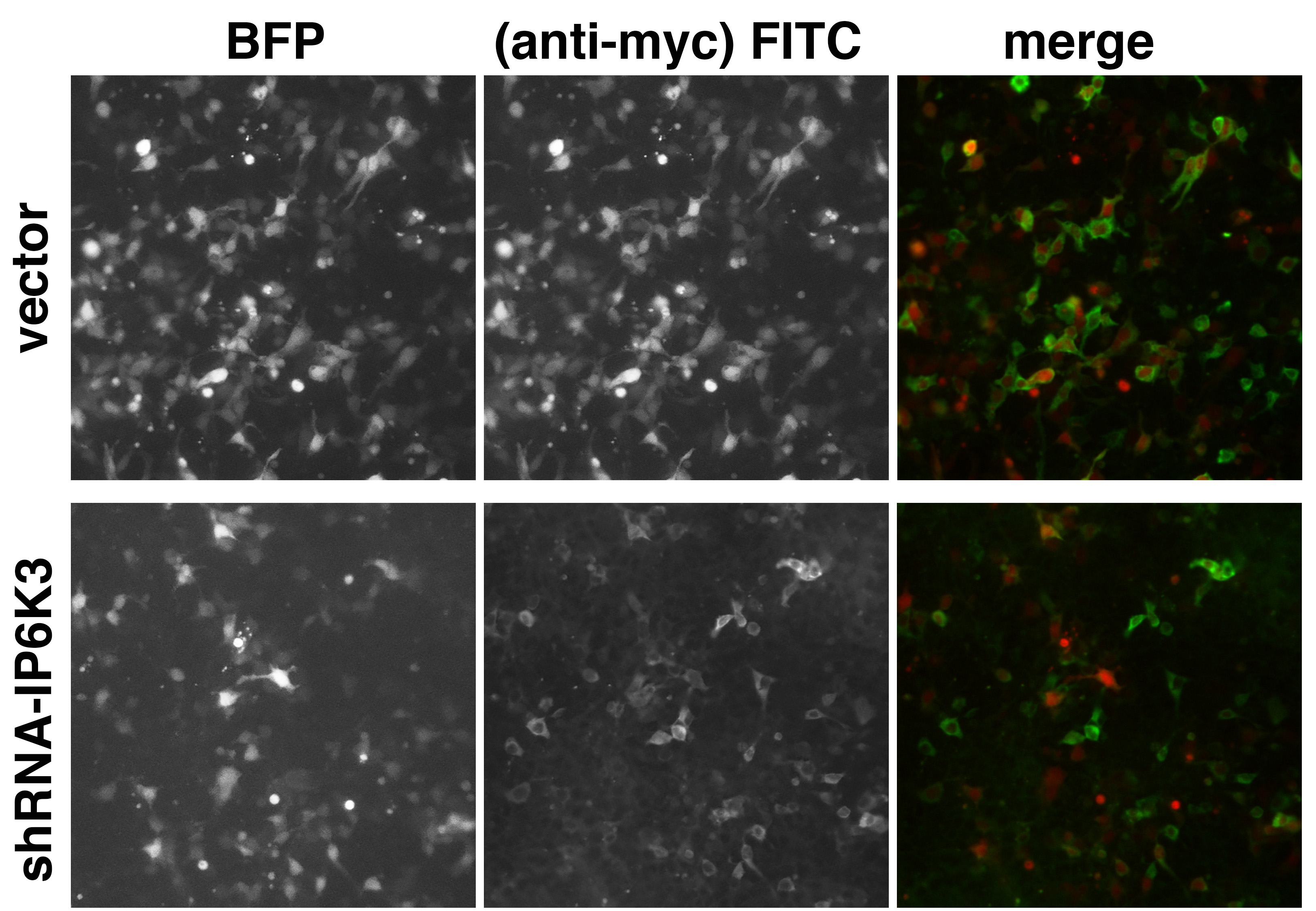

Supplement: SUPPLEMENTARY FIGURE 3 — Knockdown of overexpressed myc-IP6K3. To verify knockdown of rat IP6K3 protein with a specific shRNA, myc-tagged rat IP6K3 proteins were first overexpressed in HEK 293 cells, then infected with either FHUGW vector control or shRNA against rat IP6K3. Fluorescence intensity of the immunoreactivity (FITC) of myc-tagged rat IP6K3 was compared with vector control. Vector treated cells show co-expression of BFP and myc-tagged protein, IP6K3 shRNA infected cells show reduced FITC (myc-tagged rat IP6K3) immunoreactivity. Knockdown of overexpressed rat IP6K3 is ∼35%. [file Image_3.JPEG]
